# Anticancer ethnomedicines for cancer treatment in Taiwan

**DOI:** 10.3389/fphar.2025.1640358

**Published:** 2025-09-15

**Authors:** Chien-Yu Ko, Min-Han Chi, Jung Chao, Shyh-Shyun Huang, Hong-Zin Lee

**Affiliations:** ^1^ School of Pharmacy, China Medical University, Taichung, Taiwan; ^2^ Chinese Medicine Research Center, Department of Chinese Pharmaceutical Sciences and Chinese Medicine Resources, Master Program for Food and Drug Safety, China Medical University, Taichung, Taiwan; ^3^ Department of Food Nutrition and Health Biotechnology, Asia University, Taichung, Taiwan

**Keywords:** anticancer ethnomedicine, Asteraceae, cancer, Taiwan, traditional medicine, questionnaire

## Abstract

**Introduction:**

Cancer remains a leading global health issue and has been the leading cause of death in Taiwan for over four decades. In recent years, ethnomedicines have gained growing attention as complementary therapies in cancer treatment. However, systematic investigations linking traditional medicinal practices with cancer remain limited. This study aims to explore the types, preparation methods, and parts used of anticancer ethnomedicines and cancer types across different regions in Taiwan through ethnobotanical surveys, and to preserve and transmit traditional knowledge.

**Methods:**

Data were collected through questionnaire-based interviews with 210 participants, sampled proportionally by region, age, gender, and education based on national demographics.

**Results:**

Chi-squared tests showed that only education level significantly influenced the number of anticancer ethnomedicines mentioned (*p* < 0.001), suggesting that Taiwan’s policies promoting traditional medicine education may enhance knowledge retention. A total of 159 ethnomedicines were mentioned, with 146 plant species classified into 66 families, predominantly Asteraceae, Lamiaceae, and Fabaceae. The most commonly used plant parts were whole herba for herbaceous species, herba and radix for lianas, and leaves for trees and shrubs. Water decoction was the most frequently reported preparation method. The top ten mentioned ethnomedicines were traditionally used for a broader range of cancers than reported in the PubMed literature, especially for breast, lung, liver, and colorectal cancers, showing high consistency between traditional usage and modern scientific findings. Consensus factor analysis revealed high agreement among respondents regarding remedies for the ten most common cancers in Taiwan, except for prostate and oral cancers, possibly due to clinical treatment limitations or low questionnaire response rate. Taiwan’s cultural diversity, linguistic unity, and unique geographical environment facilitate the accurate and comprehensive collection of ethnomedicinal data, leading to more valuable research outcomes.

**Discussion:**

In summary, the results of this study provide a valuable foundation for future anticancer research, serving as a priority focus for further investigation into the underlying mechanisms of anticancer activity. Moreover, this research supports the scientific development and potential drug discovery of ethnomedicines in modern cancer treatment, contributing to the integration of traditional knowledge with contemporary biomedical approaches.

## 1 Introduction

Traditional medicine (also known as alternative medicine, unorthodox medicine or fringe medicine) has a long history of inherent theory, belief and experience of national culture. In general, natural products, spiritual therapies and operational therapies were used to prevent, diagnose or treat physical and mental health problems of traditional medicine. A variety of traditional medical practices are believed to have a special effect on diseases even though traditional medicine lacks scientific basis and empirical evidence. India’s Āyurveda, Greece and Arabias’ Unani, China’s acupuncture, South Africa’s Muti and West Africa’s Ifá are well-known traditional medicines. Among them, India’s Āyurveda is a traditional medical treatment consisting of medicinal plants, massage and yoga therapy. It is noteworthy that traditional medicine was collected in the International Classification of Diseases 11th Revision (ICD-11) in 2018, suggesting that it is of increasing importance in the global healthcare system. Humans have maintained a close relationship with plants. In addition to using plants as food sources, humans have also been using plants to treat diseases and maintain health. Archaeological evidence shows that the four ancient civilizations of Mesopotamia, ancient Egypt, ancient China, and ancient India have a large number of records on the use of various medicinal plants (Diamond, 1997; Fuller et al., 2014), suggested that plants are an integral to the survival and development of human civilization. Culturally rooted traditional medical knowledge and practices have been passed down for thousands of years. Ethnobotanical research not only helps to understand the practical applications of plants by local people but also can elucidate plant resources, traditional knowledge, and cultural practices, and even provide valuable information for new drug discovery. Therefore, ethnomedicines have played an important role in the history of drug development.

Ochwang’i et al. (2014) investigated the use of medicinal plants to treat cancer of residents in Kakamega County, Kenya, indicating that 65 anticancer plants were used by local residents, in which 25 had not been reported previously. Among the 25 anticancer plants, the most frequently mentioned is *Spathodea campanulata* Beauv. [Bignoniaceae]. The study also indicated that local residents ground the bark, roots or leaves of *S. campanulata* into powder, and then make an oral liquid with water, broth or alcohol to treat cervical, bone, breast, colorectal or skin cancer (Ochwang’i et al., 2014). According to the ethnobotanical survey results of Ochwang’i et al., the extracts of the bark and leaves of *S. campanulata* were used to explore the antioxidant, anticancer and antiviral activities by Świątek et al. (2022). Willow bark has been used by the ancient Greek physician Hippocrates to relieve pain and reduce fever before the Christian era. The German pharmacologist Johann Andreas Buchner purified salicin from willow bark in 1828, and then Italian chemist Raffaele Piria (1836) converted salicin into a sugar and a second component, which on oxidation becomes salicylic acid, a raw material for preparing the analgesic drug aspirin (acetylsalicylic acid). For reducing the side effects of gastric irritation caused by salicylic acid, German chemist Felix Hoffman synthesized aspirin by the acetylation of salicylic acid. Aspirin is a well-known analgesic, antipyretic and anti-inflammatory agent and has been used for more than 100 years. Furthermore, when Youyou Tu accepted the 2015 Nobel Prize in Physiology or Medicine, her acceptance speech was entitled, ‘Discovery of Artemisinin: A Gift from Traditional Chinese Medicine to the World‘. Therefore, it is expected to provide the authentic uses of anticancer plants and information for the development of new drugs and the traditional Chinese medicine industry through our investigations of the traditional medicines and their applications in the treatment or prevention of cancers in Taiwan.

Cancer is the second leading cause of death globally, and has been the leading cause of death in Taiwan for 42 consecutive years. The International Agency for Research on Cancer (IARC) report provides that there were 20 million new cancer cases and 9.7 million deaths globally, with lung and breast cancer being most common in 2022. According to the report provided by the Ministry of Health and Welfare in Taiwan, the 10 most common types of cancer were lung, liver, colorectal, breast, prostate, oral, pancreatic, stomach, esophageal and ovarian cancer in 2023. Due to the efforts of various countries in cancer prevention, treatment, and long-term care, cancer has become a major global public health issue and an economic burden. In general, traditional medicine is often used to relieve the symptoms caused by medication or improve treatment efficacy during cancer treatment (Lo et al., 2012; Kalender et al., 2014; Li et al., 2017; Park et al., 2019; Radha Abbas Hasoon and Jawad Kadhim, 2021). Shen-Mai-San, which is composed of *Panax ginseng* C.A.Mey. [Araliaceae; Ginseng radix et rhizoma], *Ophiopogon japonicus* (Thunb.) Ker Gawl [Liliaceae; Ophiopogonis radix] and *Schisandra chinensis* (Turcz.) Baill. [Magnoliaceae; Schisandrae fructus], was demonstrated to help reduce cancer fatigue during cancer treatment (Lo et al., 2012). It has been suggested that Nettle root or leaf can reduce anxiety and depression caused by cancer treatment (Kalender et al., 2014). *P. ginseng* was also demonstrated to reduce doxorubicin-induced cardiotoxicity and improve its anticancer activity (Li et al., 2017; Park et al., 2019; Radha Abbas Hasoon and Jawad Kadhim, 2021). Based on the above findings, traditional medicine as adjunctive therapy in cancer patients can effectively alleviate the discomfort caused by medication or improve treatment efficacy during cancer treatment when used correctly. Although traditional medicine is often used for adjuvant treatment of cancer around the world, there is still a lack of systematic investigation of the relationship between ethnobotany and cancer treatment.

The knowledge and practices of traditional medicines that have been transmitted and hybridized for thousands of years within the cultures and life systems of Han Chinese, indigenous people and foreign peoples in Taiwan that offers Taiwan’s unique traditional medical knowledge. Since small territory and high population density of Taiwan, high degree of consistency achieved regarding the efficacy of the plants among respondents from the four major administrative regions of northern, central, southern and eastern Taiwan. Taiwan has a well-developed communication and transportation system, which makes it easy to collect questionnaire data from respondents all over the country and increases the response rate of the questionnaire. There is a growing awareness of participation in social issues and public policy of Taiwanese people that will be more than willing to participate in questionnaire surveys and provide opinions and suggestions, further increasing the response rate of the questionnaire. Mandarin is Taiwan’s official language and is spoken by nearly 96.9% of Taiwan’s population. Therefore, respondents can be communicated better and answer to the questionnaire, thereby improving data accuracy. Based on the above reasons, conducting questionnaire surveys in Taiwan will be more advantageous than in other countries. The major purpose of this study was to systematically investigate and gain further understanding of the traditional medicines and their applications in the treatment or prevention of cancers in Taiwan. The results of this study can be used to further clarify the plant resources, knowledge and culture in a certain area and even provide useful information for researchers to explore the mechanism of anticancer activity in future and promote the development of new drugs and the traditional Chinese medicine industry.

## 2 Materials and methods

### 2.1 Introduction of study area

**FIGURE 1:**
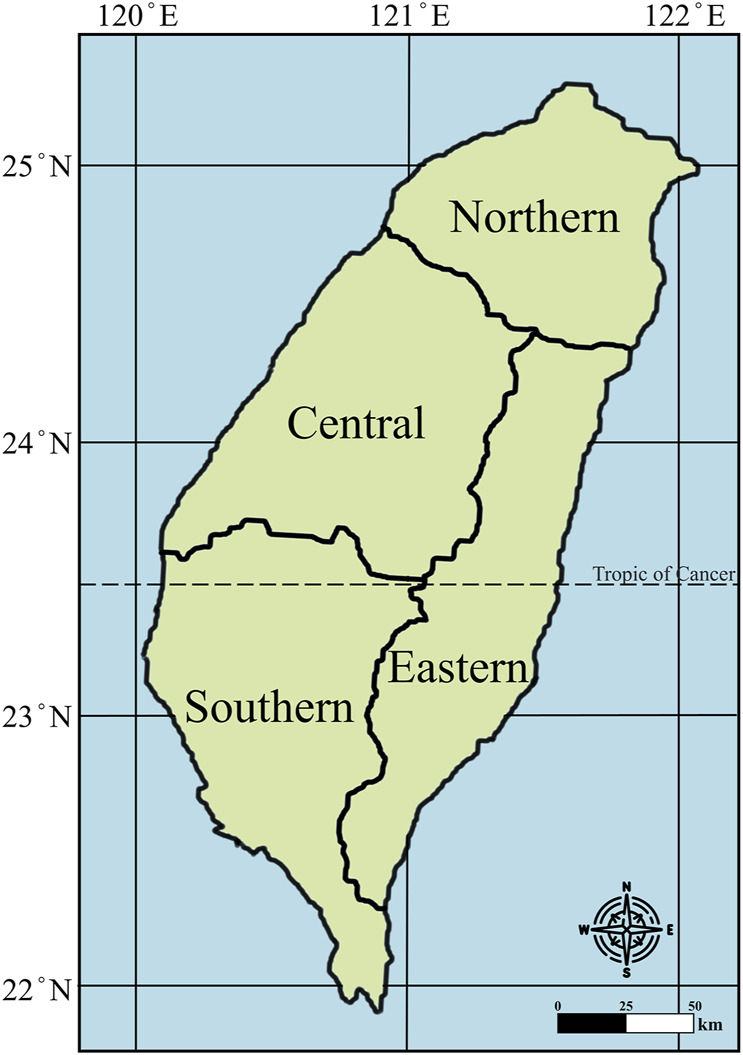
Four political geographies of Taiwan.

Taiwan is an island in East Asia, has an area of approximately 36,000 km^2^. The Central Mountain Range is the principal mountain range on the island of Taiwan. It runs from the north of the island to the south. Due to this separation, connecting between the west and east is not convenient. The terrain is mainly divided into mountains, hills, plateaus, basins and plains, with mountains and hills accounting for about 70% of the total area. The climate is humid subtropical and tropical monsoon, with high temperatures and abundant rainfall throughout the year. Taiwan enjoys an impressive forest coverage rate of 60.71% and is surrounded by the sea making it rich in natural resources. Taiwan has a population of about 23 million, and due to the topography of the country, the population is mainly located in the plains, platforms and basins of the western coastal region, and the ethnic groups are mainly divided into Han Chinese and indigenous peoples. Each ethnic group has its own culture, language, customs and social structure. In addition, Taiwan has also received foreign cultural influences from Japan, Europe, and the U.S. Because of Taiwan unique geographic location, complex topography, rich natural resources, diverse ethnic structure, and foreign cultural influences, a colorful and varied ethnic culture has been woven into the fabric of the country, which has given rise to the unique knowledge of traditional medicine in Taiwan. According to the Taiwan area comprehensive development plan formulated by the National Development Council of the Executive Yuan of Taiwan, Taiwan is divided into four major administrative regions: northern, central, southern, and eastern (Figure 1). Therefore, this study conducted field investigation and data collection based on these four administrative regions.

### 2.2 Field work and data collection

The field investigation and data collection for this study was carried out between December 2022 and December 2023 by using semi-structured questionnaire to collect data from a total of 272 respondents. However, due to incomplete data, such as the areas of residence, gender, age, education or occupation not filled, from some respondents, only 210 respondents were ultimately included in the analysis. The questionnaire was divided into two parts: basic information and ethnobotanical information. The basic information of the respondents included the areas of residence, gender, age, education and occupation, while the ethnobotanical information covered the ethnomedicine name, part of use, preparation method, and type of cancer treatment. According to statistics from the Ministry of the Interior of Taiwan, the proportions of the population over 20 years old in the four major administrative regions are 46.13%, 24.70%, 26.87% and 2.3%, respectively. In each region, the gender ratio is approximately 1:1, with a significant proportion of the population being middle-aged. Additionally, statistics indicate that approximately 40% of Taiwanese graduates have a university degree or above (including bachelor, master and doctorate degrees). Therefore, this study intends to conduct a survey based on the aforementioned population distribution ratios. In addition to collecting respondents based on the above conditions, in order to avoid bias in the survey results caused by specific occupations, this study also collected an appropriate number of questionnaires from all walks of life. This study was approved by the Research Ethics Committee Ⅱ of the China Medical University and Hospital, Protocol No. CMUH111-REC2-191, and the study was conducted on ethnic groups aged 20 years or older. Before data collection, each respondent was given a detailed explanation of the study content. Since multiple ethnomedicines may correspond to the same local name, relevant online images were immediately searched for and presented to the respondent for confirmation when an ethnomedicine was mentioned, in order to avoid misrecording the ethnomedicine. After the interview, the respondent confirmed that the content of the interview was correct and signed an informed consent form. Respondents were informed that they could withdraw from the study at any time and for any reason, and that all steps of the study, including the principles of protection, informed consent, respect for privacy, and reciprocity and feedback, were conducting according to the Declaration of Helsinki and the Code of Ethics of the International Society of Ethnobiology (ISE).

### 2.3 Biological classification of traditional anticancer medicines collected

In this study, the collected Taiwan anticancer ethnomedicines were biologically classified according to the Taxonomy by Cavalier-Smith and the World Checklist of Vascular Plants (WCVP). The Taiwan anticancer ethnomedicines organs mentioned by the respondents were confirmed by Prof. Shyh-Shyun Huang of the China Medical University. The scientific names and family names used were based on the World Flora Online (WFO), the International Code of Zoological Nomenclature (ICZN), and the International Code of Nomenclature for algae, fungi, and plants (ICN).

### 2.4 Electronic database survey

This study searched PubMed based on the scientific name of the anticancer ethnomedicines and the keyword cancer or tumor, and obtained a collection of information related to the top ten anticancer ethnomedicines in research studies on the types of cancers treated.

### 2.5 Data analysis

In this study, the results of the ethnobotanical data were analyzed using descriptive statistics which includes percentage calculation and informant consensus factor (ICF). ICF value is used to objectively assess the level of agreement on conventional treatment regimens for different cancer types, and its magnitude is directly proportional to the level of agreement on the therapeutic agents chosen by the respondents for the treatment of a specific cancer type. The following are the formulae for calculating ICF values: ICF = $\frac{N_{\text{ur}}-N_{t}}{N_{\text{ur}}-1}$ , ranges from 0 to 1, where N_ur_ denotes the number of ethnic drugs mentioned for treating each cancer type, and N_t_ denotes the number of different ethnic drugs mentioned in N_ur_.

## 3 Results

### 3.1 Characteristics of respondents

In order to investigate anticancer ethnomedicine in Taiwan, this study conducted a random questionnaire survey among 210 respondents. According to the Human Subjects Research Act promulgated by the Ministry of Health and Welfare of the Executive Yuan of Taiwan, where the subject has been judicially declared to be of limited legal capacity (over 7 years old but under 20 years old) or under assistance, consent shall be obtained from both the individual and their legal representative or assistant. Therefore, the subjects of this study are people over 20 years old. Taiwan is divided into four major administrative regions: northern, central, southern, and eastern. The proportions of the population over 20 years old in these four administrative regions are 46.13%, 24.70%, 26.87% and 2.3% respectively. Therefore, this study is also approximately conducted a questionnaire survey on the population distribution ratio (Table 1). In terms of gender distribution, although the distribution of men and women over the age of 20 in Taiwan is 48.77% and 51.23% respectively, the respondents in this study were more women (61.90%) than men (38.10%) (Table 1). During the survey, according to the demographic data of the Department of Household Registration of the Ministry of the Interior of Taiwan, the population over 20 years old was dominated by the middle-aged group (49.12%). As shown in Table 1, the age structure of the questionnaire respondents in this study is also concentrated in the middle-aged group, which is similar to the age structure of the Taiwan population. According to statistics from Taiwan Ministry of the Interior in 2023, approximately 40% of Taiwanese graduates have a university degree or above (including bachelor, master and doctorate degrees), which is close to the proportion sampled in this study (Table 1). This shows that from the perspective of education level, the sample in this study also conforms to the educational level characteristics of the Taiwan population. In addition to collecting respondents based on the above conditions, in order to avoid bias in the survey results caused by specific occupations, this study also collected an appropriate number of questionnaires from all walks of life (Table 1). The study uses the Chi-squared test to statistically analyze the correlation between the average number of anticancer ethnomedicines mentioned by different groups and the total sample. It is worth noting that in addition to different education levels, it will affect the number of anticancer ethnomedicines mentioned by the respondent, there were no significant differences in other factors such as areas of residence, gender, age, and occupations (Table 1).

**TABLE 1:** Informant mentioned anticancer ethnomedicine species characteristics.

Characteristics	N^a^	Percentage (%)^b^	Mean^c^ ± SE	*p*-value^d^
Total	210	100	5.62 ± 0.23	
Areas of residence				0.727
Northern	93	44.29	5.40 ± 0.32	
Central	55	26.19	6.04 ± 0.48	
Southern	52	24.76	5.69 ± 0.52	
Eastern	10	4.76	4.90 ± 0.78	
Gender				0.571
Male	80	38.10	6.08 ± 0.41	
Female	130	61.90	5.33 ± 0.28	
Age				0.528
21–30	21	10.00	5.62 ± 0.56	
31–40	14	6.67	6.14 ± 0.93	
41–50	56	26.67	5.63 ± 0.36	
51–60	71	33.81	6.08 ± 0.50	
61–70	27	12.86	5.56 ± 0.54	
71–80	19	9.05	3.79 ± 0.62	
>80	2	0.95	3.00 ± 2.00	
Education				<0.001
Elementary school	14	6.67	4.21 ± 0.70	
Junior high school	10	4.76	5.00 ± 1.33	
Senior high school and vocational high school	39	18.57	5.59 ± 0.44	
Junior college	43	20.48	5.51 ± 0.53	
Bachelor	63	30.00	6.13 ± 0.39	
Master	36	17.14	5.36 ± 0.66	
Doctorate	5	2.38	7.20 ± 2.29	
Occupations				0.985
Military, public, educational, state-owned enterprise personnel and professionals	70	33.33	6.13 ± 0.45	
Technicians and associate professionals	8	3.81	3.50 ± 0.68	
Clerical support workers	23	10.95	5.91 ± 0.60	
Service and sales workers	56	26.67	5.73 ± 0.47	
Skilled agricultural, forestry and fishery workers	15	7.14	5.73 ± 0.69	
Plant and machine operators, and assemblers	3	1.43	5.67 ± 0.33	
Elementary laborers	6	2.86	3.33 ± 1.20	
Others	29	13.81	5.07 ± 0.59	

^a^ N represents the respondent numbers of group.

^b^ The percentage is the numbers of group divided by the total respondents.

^c^ The mean of the numbers of anticancer ethnomedicines.

^d^ From Chi-squared test of association. ^***^ *p* < 0.001 evaluate correlations between the mean of groups and total samples.

### 3.2 Biological classification and traditional knowledge of anticancer ethnomedicines collected

**FIGURE 2:**
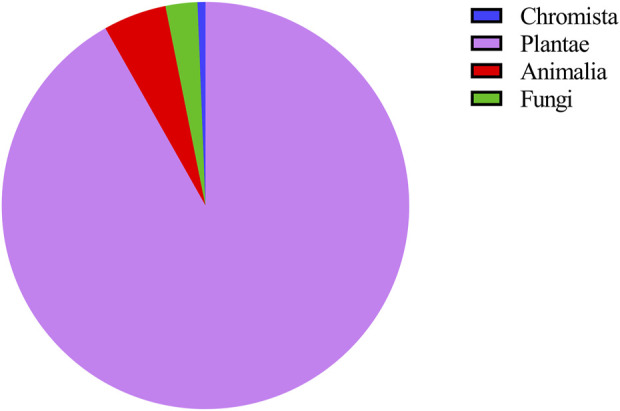
Distribution of 159 anticancer ethnomedicines in kingdom.

**FIGURE 3:**
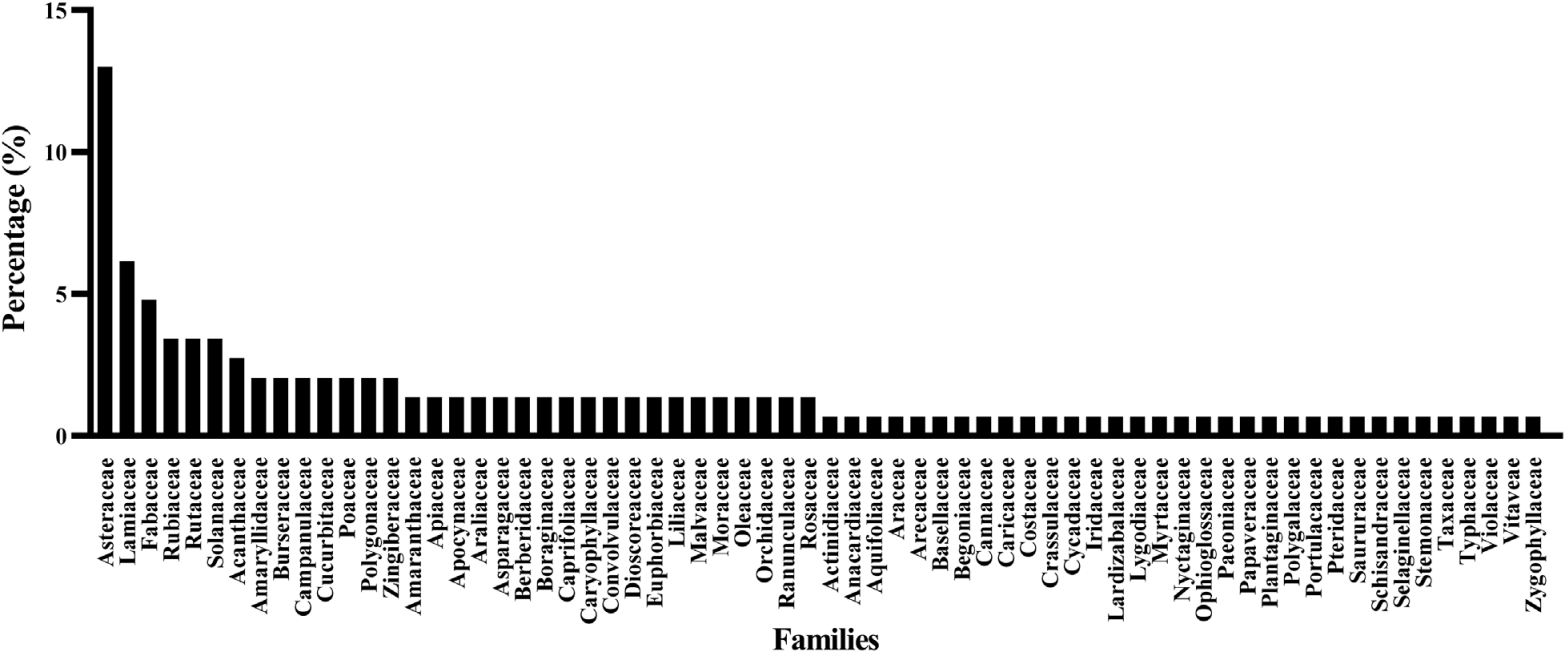
Distribution of 146 anticancer medicinal plants in family.

This study biologically classified 159 anticancer ethnomedicines collected from 210 respondents. According to the biological classification method proposed by Cavalier-Smith, organisms can be divided into seven kingdoms: Animalia, Protozoa, Fungi, Plantae, Chromista, Bacteria and Archaea. Among the 159 anticancer ethnomedicines in this study, 146 are distributed in Plantae (91.82%), 8 in Animalia (5.03%), 4 in Fungi (2.52%), and 1 in Chromista (0.63%) (Figure 2). Further, this study used the WCVP system to classify the collected anticancer medicinal plants. The 146 anticancer medicinal plants are distributed in 66 different families, including 19 species of Asteraceae (13.01%), 9 species of Lamiaceae (6.16%), and seven species of Fabaceae (4.79%) (Figure 3). Based on the above data, Asteraceae, Lamiaceae and Fabaceae plants are commonly used anticancer medicinal plants in Taiwan. This result may be related to other researchers who often use Asteraceae, Lamiaceae and Fabaceae plants for anticancer research.

**FIGURE 4:**
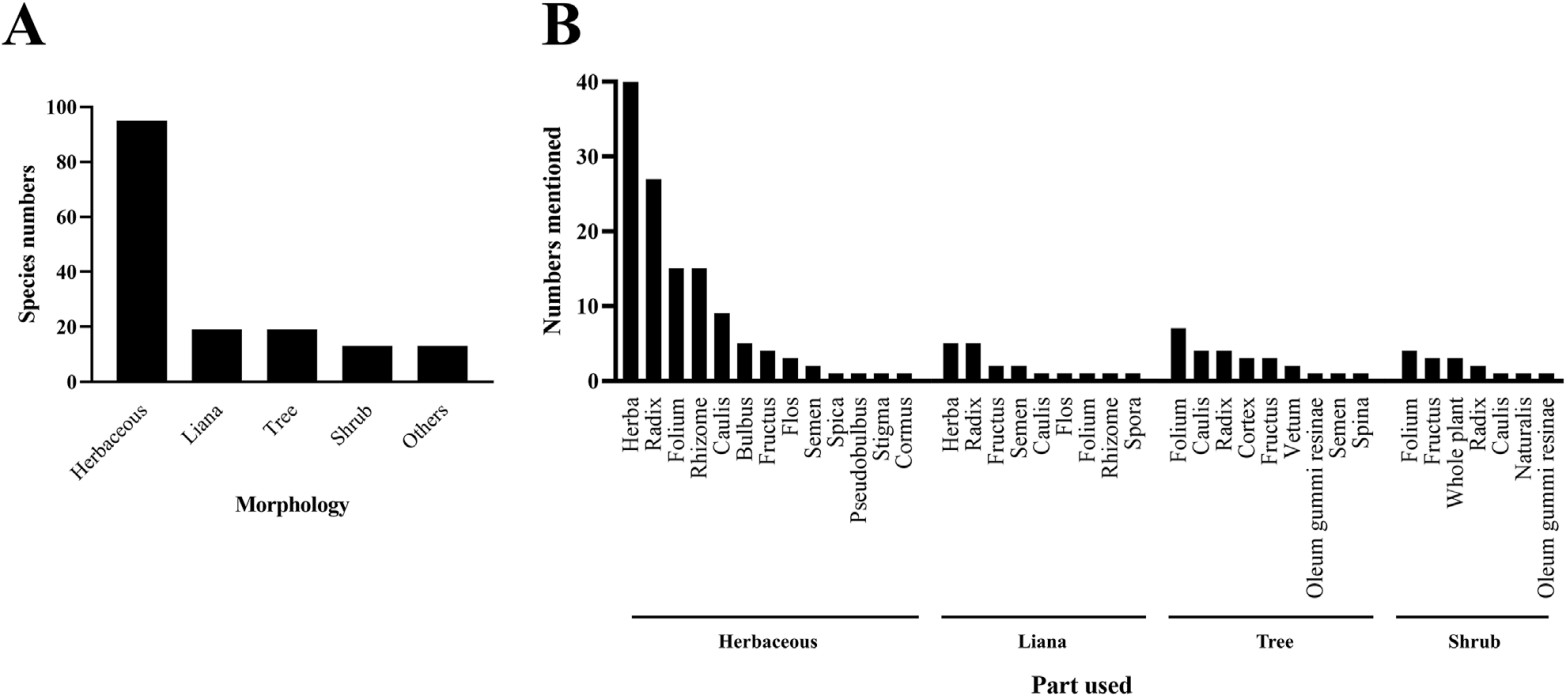
Statistics of morphology and parts used of 159 anticancer ethnomedicines. **(A)** Statistics of morphology of 159 anticancer ethnomedicines. Animalia, chromista and fungi are summarized as others. **(B)** Statistics of parts used of 159 anticancer ethnomedicines. Two medicinal parts used of the same plants were counted as two types, *etc.*

**FIGURE 5:**
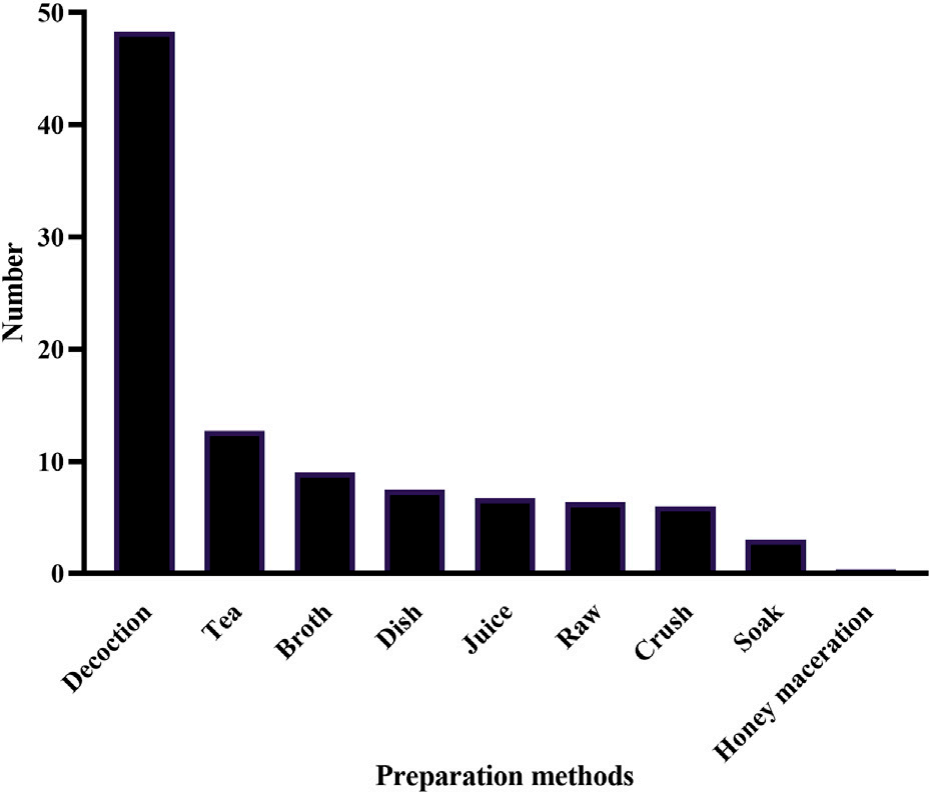
Distribution of preparation methods of 159 anticancer ethnomedicines. Two preparation methods of the same plants were counted as two types, *etc.*

In traditional medicine, different parts used and preparation methods are used to treat diseases, such as leaves, roots, fruits, seeds or flowers of plants. In terms of preparation methods, decoction, crush or soaking are used. Therefore, investigating species parts used and preparation methods will help to rationally and effectively utilize these precious biological resources. According to biomorphological classification, 159 anticancer ethnomedicines can be classified into 95 herbaceous, 19 lianas, 19 trees, 13 shrubs, and 13 others (including animals, fungi and algae) (Figure 4A; Supplementary Table S1). It can be seen from the above results that herbaceous are commonly used among people to treat cancer, which may be related to the fact that herbaceous are easy to grow and collect. Further analysis of the parts used in 146 anticancer medicinal plants shows that herbaceous mainly use herba, lianas mainly use herba and radix, and trees and shrubs mainly use folium (Figure 4B). These differences reflect the diversity and uniqueness of different plant forms in ethnomedicinal resources. In addition, the preparation methods of 159 anticancer ethnomedicines include decoction, tea, broth, dish, juice, raw, crush, soak and honey maceration (Figure 5; Supplementary Table S1). Decoction is the most common preparation method for anticancer ethnomedicine.

### 3.3 Relationship between 159 anticancer ethnomedicines collected and frequency used in cancer treatment

**FIGURE 6:**
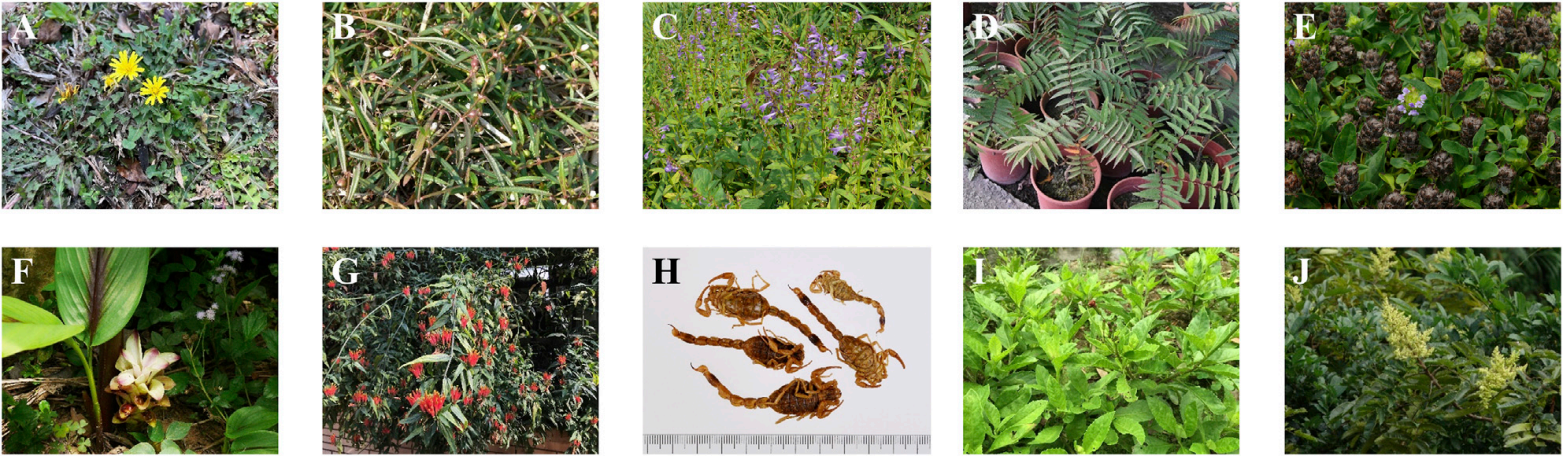
The top ten anticancer ethnomedicines. **(A)** *Taraxacum formosanum* Kitam., 64.8%; **(B)** *Scleromitrion diffusum* (Willd.) R.J.Wang, 59.5%; **(C)** *Scutellaria barbata* D. Don, 58.1%; **(D)** *Zanthoxylum ailanthoides* Siebold & Zucc., 55.2%; **(E)** *Prunella vulgaris* L., 55.2%; **(F)** *Curcuma phaeocaulis* Valeton, 39.5%; **(G)** *Clinacanthus nutans* (Burm.f.) Lindau, 34.3%; **(H)** *Buthus martensii* Karsch, 30.5%; **(I)** *Gynura procumbens* (Lour.) Merr., 17.6%; **(J)** *Rhus chinensis* var. *roxburghii* (DC.) Rehder, 14.3%.

To date, clinical medicine has discovered more than hundreds of cancers. To clarify the relationship between Taiwan folk anticancer ethnomedicines and the types of cancer treated, this study analyzed the cancer type treated by 159 anticancer ethnomedicines collected. It was found that these anticancer ethnomedicines are used in the treatment of 26 different cancer types, such as liver, colorectal, and lung cancer. In order to analyze the frequency of use of 159 anticancer ethnomedicines collected in Taiwan, the study calculated the proportion of each anticancer ethnomedicine. The proportion is directly proportional to the frequency of use of the ethnomedicine to treat cancer. The results show that the top ten anticancer ethnomedicines are *Taraxacum formosanum* Kitam. (Asteraceae), *Scleromitrion diffusum* (Willd.) R.J.Wang (Rubiaceae), *Scutellaria barbata* D. Don (Lamiaceae), *Zanthoxylum ailanthoides* Siebold & Zucc. (Rutaceae), *Prunella vulgaris* L. (Lamiaceae), *Curcuma phaeocaulis* Valeton (Zingiberaceae), *Clinacanthus nutans* (Burm.f.) Lindau (Acanthaceae), *Buthus martensii* Karsch (Buthidae), *Gynura procumbens* (Lour.) Merr. (Asteraceae) and *Rhus chinensis* var. *roxburghii* (DC.) Rehder (Anacardiaceae), their appearance is shown in Figure 6. The top ten anticancer ethnomedicines include seven herbaceous, two trees and one animal. From the results, herbaceous plants are frequently used in cancer treatment, which may be related to the fact that herbaceous are easy to grow and collect. This study further explores the differences in the number of times respondents mentioned the top ten anticancer ethnomedicines among the four major administrative regions of Taiwan: northern, central, southern and eastern Taiwan. The data is shown in Table 2, the top three mentioned by the north of respondents are *T. formosanum*, *S*. *diffusum* and *S*. *barbata*. The top three mentioned by the central of respondents are *S*. *diffusum*, *P*. *vulgaris* and *T*. *formosanum*. The top three mentioned by southern respondents are *T*. *formosanum*, *Z. ailanthoides* and *P*. *vulgaris*, while the top three mentioned by eastern respondents are *S*. *barbata*, *B. martensii*, *T*. *formosanum*, *P*. *vulgaris*, *Z*. *ailanthoides*, *C*. *phaeocaulis* and *C*. *nutans*. It can be seen from the above results that the respondents in the four major administrative regions have a consistency on the role of *T. formosanum* in cancer treatment. The results of this study also show that the total number of anticancer ethnomedicine species mentioned by respondents in the four major administrative regions are 78, 86, 66 and 10 respectively. The average number of anticancer ethnomedicine species mentioned by each respondent were 0.84, 1.56, 1.27 and 1.00 respectively. From the above results, it can be seen that the types of anticancer ethnomedicine used by respondents in central of Taiwan are more diverse.

**TABLE 2:** Top three ethnomedicines collected between various regions.

Areas of residence	1st	2nd	3rd
Northern	*Taraxacum formosanum* Kitam. (69.9%)	*Scleromitrion diffusum* (Willd.) R.J.Wang (61.3%)	*Scutellaria barbata* D.Don (59.1%)
Central	*Scleromitrion diffusum* (Willd.) R.J.Wang (61.8%)	*Prunella vulgaris* L. (56.4%)	*Taraxacum formosanum* Kitam. (54.5%) *Scutellaria barbata* D.Don (54.5%)
Southern	*Taraxacum formosanum* Kitam. (69.2%)	*Zanthoxylum ailanthoides* Siebold & Zucc. (63.5%)	*Prunella vulgaris* L. (61.5%)
Eastern	*Scutellaria barbata* D.Don (80.0%)	*Buthus martensii* Karsch (60.0%)	*Taraxacum formosanum* Kitam. (50.0%) *Prunella vulgaris* L. (50.0%) *Zanthoxylum ailanthoides* Siebold & Zucc. (50.0%) *Curcuma phaeocaulis* Valeton (50.0%) *Clinacanthus nutans* (Burm.f.) Lindau (50.0%)

### 3.4 Relationship of top ten ethnomedicines collected between traditionally used and documented researches in cancer treatment or the top ten cancers in Taiwan

The scientific value of traditional medical knowledge can be proven by analyzing the correlation between traditionally used and documented researches. To explore the relationship between the traditionally used of the top ten anticancer ethnomedicines collected and documented researches, this study used PubMed to search for literature on these ethnomedicines across different cancer types. As shown in Supplementary Table S2, the top ten collected anticancer ethnomedicines have more traditional cancer treatment types than those recorded in documented researches, covering almost all cancer types. For example, the use of *T. formosanum* in traditional anticancer treatments can treat 17 types of cancer, while documented researches literature has anticancer research reports on four types of cancer, among which liver, breast and lung cancer are the cancer types mentioned by both. It can be seen that the traditionally used of anticancer ethnomedicines and documented researches have a mutually validating relationship. The results of this study can provide an important reference for subsequent anticancer researchers and promote the innovative development of modern medical care, hoping to bring new possibilities for cancer treatment.

In recent years, traditional medicine has attracted much attention in the treatment of cancer. Ethnobotanical surveys can reveal the application experience of ethnic medicine in the treatment of various types of cancer. Generally speaking, quantitative analysis methods, such as the ICF, can be used to objectively assess the degree of consensus on traditional treatment options for different cancer types. The ICF value ranges from 0 to 1, and its size is related to the respondent’s opinion on the specific treatment. The degree of consensus on the selected treatment drug is directly proportional to the type of cancer. In order to understand the correlation between the collected anticancer ethnomedicines and cancer types, this study used the analysis ICF to understand the respondents’ consensus on the treatment of certain anticancer ethnomedicines in specific cancer types. The results show that in addition to prostate cancer and oral cancer, respondents have a high degree of consensus on the anticancer ethnomedicines used in the top ten cancers in Taiwan. In particular, breast, lung, colorectal, liver, and gastric cancer were mentioned extremely frequently (Table 3). In addition to the top ten cancers in Taiwan, lymphoma and cervical cancer also have high consensus and number of mentions among respondents (Table 3). This result shows that Taiwan traditional medicine has accumulated rich experience and consensus in the treatment of some common cancers. Ethnomedicines for the treatment of prostate cancer and oral cancer still need to be further collected and compiled to confirm that the low consensus in this study is not due to the low number of mentions by respondents. This study further explored the top three anticancer ethnomedicines collected for various types of cancer in Taiwan and found that these top three anticancer ethnomedicines were similar to the top ten anticancer ethnomedicines mentioned by the respondents (Table 4; Supplementary Table S1). Taking the treatment of breast cancer as an example, the top three anticancer ethnomedicines are *T. formosanum*, *P. vulgaris* and *S. barbata*, and these three anticancer ethnomedicines are also among the top ten anticancer ethnomedicines mentioned by the interviewees (Supplementary Table S1). In summary, the top ten anticancer ethnomedicines collected in this study have extensive anticancer application experience in Taiwan. Therefore, it is worthy of being a priority research object and anticancer activity mechanism for subsequent anticancer researchers, with a view to further developing new anticancer drugs.

**TABLE 3:** Use reports (N_ur_), number of species (N_t_) and informant consensus factor (ICF) of ethnomedicine by cancer categories in the present study.

Taiwan incidence rank	Cancer categories	Use reports (N_ur_)^a^	Number of species (N_t_)^b^	Informant consensus factor (ICF)
1	Breast cancer	179	42	0.77
2	Lung cancer	196	54	0.73
3	Colorectal cancer	211	32	0.85
4	Prostate cancer	12	8	0.36
5	Liver cancer	315	57	0.82
6	Oral cancer	18	10	0.47
7	Uterine cancer	21	9	0.60
8	Thyroid cancer	26	6	0.80
9	Ovarian cancer	12	5	0.64
10	Gastric cancer	150	29	0.81
–	Skin cancer	21	11	0.50
–	Pancreatic cancer	17	5	0.75
–	Lymphoma	84	14	0.84
–	Cervical cancer	57	14	0.77
–	Esophageal cancer	42	14	0.68
–	Leukemia	22	11	0.52
–	Nasopharyngeal cancer	18	10	0.47
–	Renal cancer	10	7	0.33
–	Bladder cancer	10	9	0.11
–	Brain cancer	6	5	0.20
–	Cholangiocarcinoma	3	2	0.50
–	Splenic cancer	2	2	0.00
–	Bone cancer	2	2	0.00
–	Small intestine cancer	1	1	0.00
–	Penile cancer	1	1	0.00
–	Pelvic cancer	1	1	0.00

^a^ The number of mentioned ethnomedicines of each cancer category.

^b^ The number of different ethnomedicines mentioned in N_ur_.

ICF = $\frac{N_{\text{ur}}-N_{t}}{N_{\text{ur}}-1}$ .

**TABLE 4:** Top three of ethnomedicines mentioned were used for top ten cancers (incidence) in Taiwan.

Taiwan incidence rank	Cancer category	Ethnomedicines (frequency, percentage)		
		1st	2nd	3rd
1	Breast	*Taraxacum formosanum* Kitam. (45, 25%)	*Prunella vulgaris* L. (31, 17%)	*Scutellaria barbata* D.Don (19, 11%)
2	Lung	*Scleromitrion diffusum* (Willd.) R.J.Wang (33, 17%)	*Scutellaria barbata* D.Don (31, 16%)	*Taraxacum formosanum* Kitam. (24, 12%)
3	Colorectal	*Zanthoxylum ailanthoides* Siebold & Zucc. (100, 47%)	*Scleromitrion diffusum* (Willd.) R.J.Wang (29, 14%)	*Taraxacum formosanum* Kitam. (24, 11%)
4	Prostate	*Rhus chinensis* var. *roxburghii* (DC.) Rehder (3, 25%) *Buthus martensii* Karsch (3, 25%)		*Taraxacum formosanum* Kitam. (1, 8%) *Scleromitrion diffusum* (Willd.) R.J.Wang (1, 8% *Scutellaria barbata* D.Don (1, 8% *Clinacanthus nutans* (Burm.f.) Lindau (1, 8%) *Euphorbia royleana* Boiss. (1, 8%) *Podophyllum pleianthum* Hance (1, 8%)
5	Liver	*Taraxacum formosanum* Kitam. (50, 16%)	*Scutellaria barbata* D.Don (49, 16%)	*Scleromitrion diffusum* (Willd.) R.J.Wang (39, 13%)
6	Oral	*Clinacanthus nutans* (Burm.f.) Lindau (4, 24%)	*Scutellaria barbata* D.Don (3, 18%)	*Scleromitrion diffusum* (Willd.) R.J.Wang (2, 12%) *Prunella vulgaris* L. (2, 12%) *Rhus chinensis* var. *roxburghii* (DC.) Rehder (2, 12%)
7	Uterine	*Curcuma phaeocaulis* Valeton (6, 27%)	*Scutellaria barbata* D.Don (5, 23%)	*Buthus martensii* Karsch (3, 14%) *Clinacanthus nutans* (Burm.f.) Lindau (3, 14%)
8	Thyroid	*Prunella vulgaris* L. (21, 81%)	*Taraxacum formosanum* Kitam. (1, 4%) *Buthus martensii* Karsch (1, 4%) *Clinacanthus nutans* (Burm.f.) Lindau (1, 4%) *Cremastra appendiculata* (D.Don) Makino (1, 4%) *Forsythia suspensa* (Thunb.) Vahl (1, 4%)	
9	Gastric	*Scleromitrion diffusum* (Willd.) R.J.Wang (31, 21%)	*Scutellaria barbata* D.Don (29, 19%)	*Buthus martensii* Karsch (18, 12%)
10	Skin	*Taraxacum formosanum* Kitam. (5, 0.24) *Buthus martensii* Karsch (5, 24%)		*Curcuma phaeocaulis* Valeton (2, 10%) *Lilium lancifolium* Thunb. (2, 10%)

## 4 Discussion

Since the rise in the global cancer incidence rate, cancer presents a serious public health problem and economic burden on society. Cancer also remained the top cause of death in Taiwan for the 42nd year last year. In recent years, ethnomedicines have garnered significant attention as adjunct therapies for cancer treatment worldwide. However, there is still a lack of systematic investigations into the relationship between ethnomedicines and cancer. Traditional medicine is a long-standing cultural practice deeply rooted in ethnic heritage. Although it often lacks sufficient scientific validation, its practical application demonstrates significant scientific value and potential. To protect and preserve the valuable traditional folk medicine culture of Taiwan, this study conducted a systematic and in-depth investigation into the types of anticancer ethnomedicines, the parts used, and their methods of application across various regions of Taiwan through ethnobotanical surveys. Based on biological taxonomy, this study found that the 159 anticancer ethnomedicines collected were mainly distributed in the herbal plants of the plant kingdom. Whole plants are primarily used for herbaceous species, while both whole plants and roots are common for climbers. For trees and shrubs, leaves are the most commonly used parts. Of the 146 medicinal plants were classified into 66 plant families using the WCVP system. The most represented families were Asteraceae (19 species), Lamiaceae (9 species), and Fabaceae (7 species). Asteraceae, the largest dicotyledon family with over 32,000 species worldwide (Achika et al., 2014; Rolnik and Olas, 2021), is known for metabolites such as essential oils, saponins, polyphenols, and polysaccharides—many of which possess anticancer, antioxidant, and anti-inflammatory properties (Yuan et al., 2012; Rolnik and Olas, 2021). Lamiaceae, the fifth-largest dicot family with more than 7,000 species (Chrysargyris, 2024), is rich in terpenoids and polyphenols that contribute to various biological activities, including anticancer effects (Wink, 2003; De Sousa et al., 2004; Stanojevic et al., 2010; De et al., 2011; Lin et al., 2012; Kamdem et al., 2013; Pop et al., 2013). Fabaceae, the third-largest dicot family comprising around 20,000 species (Lewis, 2005), contains abundant polyphenols and proteins with demonstrated anti-inflammatory, antioxidant, and antiancer activities (Boudjou et al., 2013; Del Carmen Millán-Linares et al., 2014; Giusti et al., 2017). The predominance of these three families among the collected anticancer medicinal plants may be due to their global distribution, prevalence of herbaceous species, ease of cultivation and harvesting, and diverse bioactive metabolites. Therefore, the present findings can be used for future research to explore related species within these families to discover more anticancer agents and potentially develop new therapeutic drugs.

This is the first ethnobotanical investigation focused on the use of traditional Taiwanese medicines in cancer treatment, incorporating responses from individuals of varying regions, genders, ages, education levels, and occupations. In the current study, 38.10% of respondents were male and 61.90% were female. The average number of anticancer ethnomedicines mentioned was 6.08 ± 0.41 for males and 5.33 ± 0.28 for females. Despite this apparent difference, statistical analysis using the Chi-squared test showed that the result was not statistically significant (*p*-value 0.571), suggesting no meaningful gender-based difference in this context. Notably, among the factors examined in this study, only education level showed a significant impact on the number of anticancer ethnomedicines mentioned by respondents. However, findings from other regions present a contrasting perspective. For example, Quinlan and Quinlan (2007) observed that each additional year of education in the Caribbean correlated with a 2.52 species decrease in ethnobotanical knowledge. Similarly, Voeks and Leony (2004) in Brazil and Lozada et al. (2006) in Argentina found that increased formal schooling and the introduction of standard biomedical treatment contributed to the erosion of traditional knowledge dissemination. In contrast, the current study suggests that education in Taiwan does not diminish traditional medicinal knowledge. This may be partly due to the implementation of Taiwan’s *Chinese Medicine and Pharmacy Development Act* in 2009, which promotes traditional medicine education as part of its sustainable development strategy. Taken together, these findings suggest that the relationship between education level and knowledge of ethnomedicine may vary significantly across different cultural and national contexts. Furthermore, with the increasing number of international publications in fields such as ethnobotany, ethnopharmacology and ethnochemistry, and the integration of scientific research helps validate the efficacy of traditional practices, thereby increasing public awareness and attention to the value of preserving cultural knowledge.

As shown in Supplementary Table S1, the 159 anticancer ethnomedicines collected in this study are traditionally used to treat 26 different types of cancer. The top ten mentioned ethnomedicines by respondents include *T. formosanum*, *S. diffusum*, *S. barbata*, *Z. ailanthoides*, *P. vulgaris*, *C. phaeocaulis*, *C. nutans*, *B. martensii*, *G. procumben* and *R. chinensis*. Among these, *T. formosanum* emerged as the most commonly recommended anticancer ethnomedicine across all four major regions of Taiwan, including north, central, south, and east. The top ten anticancer ethnomedicines in traditional use were applied to a wider range of cancer types than those reported in the PubMed scientific literature and covered nearly all cancer types documented in those studies. In particular, there is a high degree of consistency and mutual validation between the traditional use of these ethnomedicines and modern cancer research in treating common cancers such as breast, lung, colorectal, and liver cancer. As shown in Supplementary Table S2, *T. formosanum* is traditionally used in Taiwan to treat 17 types of cancer. However, only four of these cancers have been investigated in PubMed literature, with liver, breast, and lung cancers being common to both lists. A member of the Asteraceae family, *T. formosanum*, an endemic species of Taiwan, contains bioactive metabolites such as luteolin, quercetin, hesperetin and stigmasterol, which have demonstrated antioxidant, anti-inflammatory, and anticancer properties (Chien et al., 2018; Lin et al., 2022a; Fan et al., 2023). A PubMed database search for studies on the component analysis and anticancer mechanisms of *T*. *formosanum* revealed the following: The study on the anticancer activity of whole plant alcoholic extracts of *T. formosanum* by inhibiting the activation of extracellular signal-regulated kinase in non-small cell lung cancer cells has been demonstrated by Chien et al. (2018). Lin et al. (2022a) and (2022b) reported that aqueous extracts from the whole plants of *T*. *formosanum* induce apoptosis in HeLa human cervical cancer cells *via* endoplasmic reticulum stress, and they also suggested that these extracts can induce ribotoxic stress on breast cancer cells. Loh et al. (2012) and Kao et al. (2012) isolated chlorophylls and carotenoids, respectively, from *T*. *formosanum*. Furthermore, Leu et al. (2003) identified three new metabolites—taraxacine-A, taraxacine-B, and taraxafolin. However, there is currently no direct evidence linking these isolated metabolites (chlorophylls, carotenoids, taraxacine-A, taraxacine-B, and taraxafolin) to the anticancer activity of *T*. *formosanum*. Our findings provide a valuable foundation for future studies on anticancer ethnomedicines. They can guide investigations into chemical composition, pharmacological mechanisms, and clinical efficacy, potentially fostering innovation in cancer therapy and contributing to the development of new treatment options. However, alignment of traditional knowledge and scientific investigations currently show limitations in insufficient methods of transmission and documentation, lack of standardization and quantification, and challenges in scientific empirical research methods such as biodiversity and sustainability, and data collection and validation.

This study employed the ICF to assess the level of agreement among respondents regarding the ethnomedicinal treatments used for various types of cancer. Except for prostate and oral cancers, there was a high degree of consensus on treatments for the top ten most prevalent cancers in Taiwan, particularly breast, lung, colorectal, liver, and stomach cancers, which were mentioned most frequently. These findings suggest that traditional medicine in Taiwan has developed substantial collective knowledge and agreement in managing several common cancer types. Prostate and oral cancers showed lower levels of consensus in this study. In Taiwan, about 80% of prostate cancer cases are localized, with treatment largely based on cancer stage, ranging from active surveillance to surgery or other therapies (Mazhar and Waxman, 2002; Sekhoacha et al., 2022). Oral cancer includes various tumors in the oral cavity, typically treated with surgery, radiation, and chemotherapy (Montero and Patel, 2015). Due to the limited effectiveness of drug therapies, surgery remains the main approach for both cancers, which may explain the lower consensus on ethnomedicinal treatments. Further investigation and documentation of traditional knowledge related to prostate and oral cancer treatments are needed to determine whether the low consensus is due to a lack of traditional practices or simply limited respondent recall. This study also found that the most commonly used ethnomedicinal treatments for various types of Taiwan’s top ten cancers were almost all included among the top ten anticancer ethnomedicines mentioned by respondents. These frequently used medicinal plants may possess significant anticancer activity and are worth prioritizing in future research to further explore their mechanisms of action and support the development of new anticancer drugs.

In summary, this study investigates the ethnomedicines used in Taiwan for cancer treatment and their methods of use. In addition to finding out the application experiences of ethnomedicines in treating various types of cancer through ethnobotanical surveys, it also plays a vital role in enhancing the understanding, preservation, and transmission of Taiwan’s rich traditional medical practices and natural resources. The study further examines the relationship between traditional folk knowledge and modern scientific research. This result highlights the scientific value of traditional medicine in cancer treatment, providing a valuable foundation for future anticancer research, serving as a priority focus for further investigation into anticancer mechanisms and supporting the scientific advancement and drug development of ethnomedicines for cancer treatment.

## Data Availability

The original contributions presented in the study are included in the article/Supplementary Material, further inquiries can be directed to the corresponding authors.
