# Supplementary material for: Anticancer ethnomedicines for cancer treatment in Taiwan
Source: Front Pharmacol. 2025 Sep 15;16:1640358. doi: 10.3389/fphar.2025.1640358 (PMC12476902; doi:10.3389/fphar.2025.1640358)
Supplement: Supplementary file 1 [file Table1.docx]

Supplementary Data

| **Table S1.** **Biological classification and traditional knowledge of anticancer ethnomedicines collected.** | | | | | | | | | |
| --- | --- | --- | --- | --- | --- | --- | --- | --- | --- |
| **Frequency^a^** | **Percentage** | **Scientific name** | **Local name** | **Kingdom** | **Family** | **Morphology** | **Use of parts** | **Preparation methods** | **Cancer type** |
| 136 | 64.8 | *Taraxacum formosanum* Kitam. | 臺灣蒲公英、蒲公英 | Plantae | Asteraceae | herbaceous | herba, radix, folium, caulis, flos | decoction, tea, broth, raw, crush, juice, dish, soak | liver cancer, breast cancer, lung cancer, colorectal cancer, skin cancer, anticancer**^b^**, gastric cancer, pancreatic cancer, various cancer**^c^**, esophageal cancer, leukemia, prostate cancer, thyroid cancer, lymphoma, ovarian cancer, uterine cancer, nasopharyngeal cancer, renal cancer, bladder cancer |
| 125 | 59.5 | *Scleromitrion diffusum* (Willd.) R.J.Wang | 白花蛇舌草 | Plantae | Rubiaceae | herbaceous | herba, folium, radix, caulis | decoction, tea, broth, juice, crush, soak, dish | liver cancer, lung cancer, gastric cancer, colorectal cancer, anticancer**^b^**, various cancers**^c^**, breast cancer, esophageal cancer, lymphoma, cervical cancer, leukemia, oral cancer, prostate cancer, pancreatic cancer, uterine cancer, bladder cancer, splenic cancer, small intestine cancer |
| 122 | 58.1 | *Scutellaria barbata* D.Don | 半枝蓮、並頭草 | Plantae | Lamiaceae | herbaceous | herba, folium, caulis | decoction, tea, broth, crush, dish, juice, honey maceration | liver cancer, lung cancer, gastric cancer, breast cancer, anticancer**^b^**, colorectal cancer, various cancers**^c^**, esophageal cancer, uterine cancer, leukemia, nasopharyngeal cancer, oral cancer, prostate cancer, pancreatic cancer, lymphoma |
| 116 | 55.2 | *Zanthoxylum ailanthoides* Siebold & Zucc. | 食茱萸、紅刺蔥、樗葉花椒 | Plantae | Rutaceae | tree | caulis, radix, folium, fructus | decoction, soak, broth, tea, dish, crush, raw | colorectal cancer, anticancer**^b^**, liver cancer, lung cancer, gastric cancer, uterine cancer |
| 116 | 55.2 | *Prunella vulgaris* L. | 夏枯草 | Plantae | Lamiaceae | herbaceous | herba, spica, flos, folium, caulis, radix | decoction, tea, broth, crush, juice, dish, raw | lymphoma, breast cancer, liver cancer, thyroid cancer, gastric cancer, anticancer**^b^**, lung cancer, esophageal cancer, leukemia, colorectal cancer, cholangiocarcinoma, oral cancer, various cancers**^c^**, cervical cancer, nasopharyngeal cancer, skin cancer, uterine cancer, brain cancer, bone cancer |
| 83 | 39.5 | *Curcuma phaeocaulis* Valeton | 莪朮 | Plantae | Zingiberaceae | herbaceous | rhizome, herba | decoction, crush, raw, tea, soak | cervical cancer, liver cancer, gastric cancer, anticancer**^b^**, uterine cancer, ovarian cancer, lung cancer, lymphoma, esophageal cancer, bladder cancer, skin cancer, colorectal cancer, leukemia, various cancers**^c^**, breast cancer, nasopharyngeal cancer, brain cancer, splenic cancer |
| 72 | 34.3 | *Clinacanthus nutans* (Burm.f.) Lindau | 憂遁草、沙巴蛇草、鱷嘴花 | Plantae | Acanthaceae | herbaceous | herba, folium, caulis | decoction, juice, tea, raw, broth, dish, soak | lymphoma, anticancer**^b^**, liver cancer, breast cancer, lung cancer, various cancers**^c^**, oral cancer, renal cancer, colorectal cancer, cervical cancer, gastric cancer, brain cancer, ovarian cancer, uterine cancer, prostate cancer, thyroid cancer, esophageal cancer, leukemia, bladder cancer |
| 64 | 30.5 | *Buthus martensii* Karsch | 蠍子 | Animalia | Buthidae | animal | corpus | decoction, crush, broth, soak, tea | liver cancer, gastric cancer, breast cancer, lung cancer, colorectal cancer, skin cancer, anticancer**^b^**, various cancers**^c^**, esophageal cancer, prostate cancer, uterine cancer, lymphoma, thyroid cancer, cervical cancer, brain cancer |
| 37 | 17.6 | *Gynura procumbens* (Lour.) Merr. | 尼基羅草 | Plantae | Asteraceae | herbaceous | folium, caulis, herba, radix | decoction, tea, juice, dish, raw, broth, crush | liver cancer, anticancer**^b^**, lung cancer, gastric cancer, various cancers**^c^**, colorectal cancer, |
| 30 | 14.3 | *Rhus chinensis* var. *roxburghii* (DC.) Rehder | 埔鹽、羅氏鹽膚木 | Plantae | Anacardiaceae | tree | radix, folium, caulis, fructus | decoction, soak, tea, broth, crush, dish, juice, raw | anticancer**^b^**, lung cancer, colorectal cancer, gastric cancer, lymphoma, nasopharyngeal cancer, prostate cancer, oral cancer, breast cancer, liver cancer, renal cancer |
| 11 | 5.2 | *Solanum nigrum* L. | 龍葵、黑子仔菜 | Plantae | Solanaceae | herbaceous | folium, fructus | decoction, dish, broth, tea | liver cancer, lung cancer, anticancer**^b^**, breast cancer, gastric cancer, esophageal cancer |
| 9 | 4.3 | *Houttuynia cordata* Thunb. | 魚腥草、臭瘥草、狗貼耳 | Plantae | Saururaceae | herbaceous | herba, caulis | decoction, juice, tea | lung cancer, anticancer**^b^**, colorectal cancer, liver cancer |
| 9 | 4.3 | *Taiwanofungus camphoratus* (M. Zang&C.H. Su) Sheng H. Wu,Z.H. Yu,Y.C. Dai&C.H. Su | 牛樟芝 | Fungi | Polyporaceae | fungi | corporis | decoction, crush, tea | liver cancer, various cancers**^c^**, colorectal cancer, esophageal cancer, ovarian cancer, brain cancer |
| 8 | 3.8 | *Astragalus mongholicus* Bunge | 黃耆 | Plantae | Fabaceae | herbaceous | radix | decoction, broth, crush | nasopharyngeal cancer, breast cancer, anticancer**^b^**, lung cancer, liver cancer |
| 7 | 3.3 | *Euphorbia royleana* Boiss. | 金剛纂、火巷、霸王鞭 | Plantae | Euphorbiaceae | shrub | caulis, folium | decoction, broth, dish | lung cancer, liver cancer, various cancers**^c^**, colorectal cancer, prostate cancer, gastric cancer |
| 7 | 0.033 | *Vernonia amygdalina* Delile | 南非葉、扁桃葉斑鳩菊 | Plantae | Asteraceae | shrub | folium | decoction, tea, juice, raw | liver cancer, anticancer**^b^**, colorectal cancer, breast cancer, renal cancer |
| 6 | 2.9 | *Cremastra appendiculata* (D.Don) Makino | 山慈菇 | Plantae | Orchidaceae | herbaceous | pseudobulbus | decoction | lung cancer, gastric cancer, breast cancer, liver cancer, esophageal cancer, thyroid cancer |
| 5 | 2.4 | *Elephantopus scaber* L. | 天芥菜、丁豎杇、地膽草 | Plantae | Asteraceae | herbaceous | herba | decoction | breast cancer, skin cancer |
| 5 | 2.4 | *Lonicera japonica* Thunb. | 金銀花、毛忍冬 | Plantae | Caprifoliaceae | liana | flos | decoction, tea | anticancer**^b^**, various cancers**^c^**, breast cancer |
| 5 | 2.4 | *Ajuga integrifolia* Buch.-Ham. | 散血草、白馬蜈蚣 | Plantae | Lamiaceae | herbaceous | herba | decoction, juice | anticancer**^b^**, colorectal cancer, liver cancer, oral cancer, nasopharyngeal cancer |
| 5 | 2.4 | *Taxus brevifolia* Nutt. | 紫杉 | Plantae | Taxaceae | tree | cortex | decoction | breast cancer, ovarian cancer |
| 5 | 2.4 | *Agrimonia pilosa* Ledeb. | 龍芽草、仙鶴草 | Plantae | Rosaceae | herbaceous | folium | decoction, tea | lung cancer, liver cancer, gastric cancer, pancreatic cancer, cervical cancer, nasopharyngeal cancer |
| 4 | 1.9 | *Ganoderma lucidum* (Leyss. ex Fr.) Karst. | 靈芝 | Fungi | Polyporaceae | fungi | corporis | decoction, tea | anticancer**^b^**, breast cancer, liver cancer |
| 4 | 1.9 | *Catharanthus roseus* (L.) G.Don | 長春花、日日春 | Plantae | Apocynaceae | herbaceous | herba, folium | decoction, raw | liver cancer, lung cancer, colorectal cancer, breast cancer, gastric cancer, leukemia |
| 4 | 1.9 | *Coptis chinensis* Franch. | 黃連 | Plantae | Ranunculaceae | herbaceous | rhizoma | decoction, crush, tea | various cancers**^c^**, lung cancer, oral cancer, leukemia |
| 4 | 1.9 | *Panax ginseng* C.A.Mey. | 人參 | Plantae | Araliaceae | herbaceous | radix, rhizoma | decoction, raw, tea, soak | lung cancer, colorectal cancer, lymphoma, renal cancer |
| 4 | 1.9 | *Andrographis paniculata* (Burm.f.) Nees | 穿心蓮 | Plantae | Acanthaceae | herbaceous | herba | decoction | liver cancer, anticancer**^b^**, lung cancer, colorectal cancer, breast cancer |
| 4 | 1.9 | *Portulaca oleracea* L. | 馬齒莧、豬母乳 | Plantae | Portulacaceae | herbaceous | herba, folium | decoction, broth | liver cancer, anticancer**^b^**, colorectal cancer, gastric cancer, bladder cancer |
| 3 | 1.4 | *Actinidia arguta* Miq. | 藤梨根、獼猴梨根、獼猴桃根 | Plantae | Actinidiaceae | herbaceous | radix | decoction | breast cancer, anticancer |
| 3 | 1.4 | *Anredera cordifolia* (Ten.) Steenis | 藤三七 | Plantae | Basellaceae | liana | rhizoma | decoction, dish | liver cancer, breast cancer |
| 3 | 1.4 | *Cirsium japonicum* DC. | 大薊 | Plantae | Asteraceae | herbaceous | radix | decoction | anticancer**^b^**, various cancers**^c^**, lung cancer |
| 3 | 1.4 | *Curcuma longa* L. | 薑黃 | Plantae | Zingiberaceae | herbaceous | rhizoma | crush, decoction, dish | anticancer**^b^**, various cancers**^c^**, uterine cancer |
| 3 | 1.4 | *Plectranthus amboinicus* (Lour.) Spreng. | 到手香 | Plantae | Lamiaceae | herbaceous | herba | decoction | anticancer**^b^**, breast cancer, liver cancer |
| 3 | 1.4 | *Crassocephalum crepidioides* S.Moore | 昭和草 | Plantae | Asteraceae | herbaceous | caulis, folium | decoction, dish | anticancer**^b^**, breast cancer, skin cancer |
| 3 | 1.4 | *Symphytum officinale* L. | 康復力 | Plantae | Boraginaceae | herbaceous | herba | decoction, juice | liver cancer, anticancer**^b^** |
| 2 | 1.0 | *Asparagus officinalis* L. | 蘆筍 | Plantae | Asparagaceae | herbaceous | herba | juice | various cancers**^c^**, lung cancer, skin cancer |
| 2 | 1.0 | *Canavalia gladiata* (Jacq.) DC. | 白鳳豆 | Plantae | Fabaceae | liana | semen | broth | anticancer**^b^**, various cancer |
| 2 | 1.0 | *Salvia chinensis* Benth. | 石見穿 | Plantae | Lamiaceae | herbaceous | herba | decoction | anticancer**^b^** |
| 2 | 1.0 | *Scolopendra subspinipes* mutilans L. Koch | 蜈蚣 | Animalia | Scolopendridae | animal | corpus | decoction | anticancer**^b^**, breast cancer |
| 2 | 1.0 | *Bos taurus* domesticus Gmelin | 牛黃 | Animalia | Bovidae | animal | calculus | crush, decoction | various cancers**^c^**, breast cancer |
| 2 | 1.0 | *Paeonia lactiflora* Pall. | 白芍 | Plantae | Nyctaginaceae | herbaceous | radix | broth, decoction | anticancer**^b^**, breast cancer |
| 2 | 1.0 | *Scutellaria baicalensis* Georgi | 黃芩 | Plantae | Paeoniaceae | herbaceous | radix | decoction | breast cancer, liver cancer |
| 2 | 1.0 | *Panax notoginseng* (Burkill) F.H.Chen | 三七 | Plantae | Lamiaceae | herbaceous | radix, rhizoma | decoction | anticancer**^b^**, esophageal cancer |
| 2 | 1.0 | *Crocus sativus* L. | 番紅花、西紅花、藏紅花 | Plantae | Araliaceae | herbaceous | stigma | decoction | various cancers**^c^**, cervical cancer |
| 2 | 1.0 | *Glycyrrhiza uralensis* Fisch. | 甘草 | Plantae | Iridaceae | herbaceous | radix, rhizoma | decoction | lung cancer, gastric cancer, pancreatic cancer |
| 2 | 1.0 | *Selaginella doederleinii* Hieron. | 石上柏、生根卷柏 | Plantae | Fabaceae | tree | caulis, folium | broth | lung cancer, colorectal cancer |
| 2 | 1.0 | *Angelica sinensis* (Oliv.) Diels | 當歸 | Plantae | Selaginellaceae | herbaceous | radix | broth, decoction | breast cancer |
| 2 | 1.0 | *Persicaria orientalis* (L.) Spach | 水紅花子 | Plantae | Apiaceae | herbaceous | fructus | decoction | lung cancer, colorectal cancer, liver cancer, gastric cancer, pelvic cancer |
| 2 | 1.0 | *Pinellia ternata* (Thunb.) Makino | 半夏 | Plantae | Liliaceae | herbaceous | rhizoma | decoction | anticancer**^b^**, gastric cancer |
| 2 | 1.0 | *Adenostemma lavenia* (L.) Kuntze | 下田菊、麻糬糊 | Plantae | Polygonaceae | herbaceous | herba | decoction, tea | lung cancer |
| 2 | 1.0 | *Lycium chinense* Mill. | 枸杞子 | Plantae | Araceae | shrub | folium, fructus | decoction, tea | liver cancer |
| 2 | 1.0 | *Tithonia diversifolia* (Hemsl.) A.Gray | 五爪金英 | Plantae | Convolvulaceae | herbaceous | herba | decoction, tea | liver cancer, lymphoma |
| 2 | 1.0 | *Allium macrostemon* Bunge | 薤白、蕗蕎 | Plantae | Asteraceae | herbaceous | bulbus | decoction, dish, raw | lung cancer, colorectal cancer |
| 2 | 1.0 | *Zanthoxylum nitidum* DC. | 崖椒 | Plantae | Solanaceae | liana | herba | decoction | lung cancer, liver cancer |
| 2 | 1.0 | *Lilium lancifolium* Thunb. | 百合 | Plantae | Cannaceae | herbaceous | bulbus | broth, decoction | various cancers**^c^**, cervical cancer, leukemia, splenic cancer |
| 2 | 1.0 | *Mirabilis jalapa* L. | 紫茉莉 | Plantae | Asteraceae | herbaceous | radix | decoction | gastric cancer, lung cancer, splenic cancer |
| 2 | 1.0 | *Canna indica* L. | 美人蕉 | Plantae | Amaryllidaceae | herbaceous | caulis | decoction | anticancer**^b^**, prostate cancer |
| 2 | 1.0 | *Ipomoea batatas* (L.) Lam. | 番薯、地瓜 | Plantae | Rutaceae | herbaceous | folium | juice, tea | lung cancer, breast cancer |
| 2 | 1.0 | *Cycas revoluta* Thunb. | 蘇鐵 | Plantae | Cycadaceae | shrub | folium | decoction | anticancer**^b^**, lung cancer, colorectal cancer |
| 2 | 1.0 | *Bidens pilosa* L. | 咸豐草、恰查某 | Plantae | Asteraceae | herbaceous | herba | broth, dish | liver cancer, prostate cancer |
| 1 | 0.5 | *Atractylodes macrocephala* Koidz. | 白朮 | Plantae | Asteraceae | herbaceous | rhizoma | decoction | liver cancer, bladder cancer |
| 1 | 0.5 | *Tomophagus colossus* | 棺菇、鬆芝 | Fungi | Ganodermataceae | fungi | corporis | decoction | various cancers**^c^** |
| 1 | 0.5 | *Rattus norvegicus* Berkenhout | 未睜眼初生幼鼠 | Animalia | Muridae | animal | caro | raw | anticancer**^b^** |
| 1 | 0.5 | *Maclura tricuspidata* Carrière | 柘木 | Plantae | Moraceae | tree | cortex | decoction | anticancer**^b^** |
| 1 | 0.5 | *Chelidonium majus* L. | 白屈菜 | Plantae | Papaveraceae | herbaceous | herba | decoction | anticancer**^b^** |
| 1 | 0.5 | *Galium spurium* L. | 豬殃殃 | Plantae | Rubiaceae | herbaceous | herba | decoction, juice | anticancer**^b^** |
| 1 | 0.5 | *Triticum aestivum* L. | 小麥苗 | Plantae | Poaceae | herbaceous | herba | juice | various cancers**^c^** |
| 1 | 0.5 | *Spatholobus suberectus* Dunn | 雞血藤 | Plantae | Fabaceae | liana | caulis | broth | liver cancer, cervical cancer |
| 1 | 0.5 | *Coix lacryma-jobi* var. *ma-yuen* (Rom.Caill.) Stapf | 薏苡仁 | Plantae | Poaceae | herbaceous | semen | broth, decoction | breast cancer |
| 1 | 0.5 | *Viola philippica* Cav. | 紫花地丁 | Plantae | Violaceae | herbaceous | herba | decoction | breast cancer |
| 1 | 0.5 | *Epimedium sagittatum* Maxim. | 淫羊藿 | Plantae | Berberidaceae | herbaceous | folium | decoction | breast cancer |
| 1 | 0.5 | *Reynoutria multiflora* (Thunb.) Moldenke | 何首烏 | Plantae | Polygonaceae | liana | folium | decoction | breast cancer |
| 1 | 0.5 | *Sargassum pallidum* (Turner) C.Agardh | 海藻 | Chromista | Sargassaceae | seaweed | sargassum | decoction | breast cancer |
| 1 | 0.5 | *Solenognathus hardwickii* (Gray) | 海龍 | Animalia | Syngnathidae | animal | corpus | decoction | breast cancer |
| 1 | 0.5 | *Commiphora myrrha* (T.Nees) Engl. | 沒藥 | Plantae | Burseraceae | tree | oleum gummi resinae | decoction | breast cancer |
| 1 | 0.5 | *Boswellia carteri* Birdw. | 乳香 | Plantae | Burseraceae | shrub | oleum gummi resinae | decoction | breast cancer |
| 1 | 0.5 | *Moschus berezovskii* Flerov | 麝香 | Animalia | Cervidae | animal | secretiones | decoction | breast cancer |
| 1 | 0.5 | *Vaccaria hispanica* (Mill.) Rauschert | 王不留行 | Plantae | Caryophyllaceae | herbaceous | semen | decoction | breast cancer |
| 1 | 0.5 | *Ilex cornuta* Lindl. & Paxton | 枸骨葉 | Plantae | Aquifoliaceae | tree | folium | decoction | lung cancer, gastric cancer, renal cancer, bone cancer |
| 1 | 0.5 | *Anoectochilus roxburghii* (Wall.) Lindl. | 金線蓮 | Plantae | Orchidaceae | herbaceous | herba | decoction | anticancer**^b^** |
| 1 | 0.5 | *Pseudostellaria heterophylla* (Miq.) Pax | 太子參 | Plantae | Caryophyllaceae | herbaceous | radix | decoction | anticancer**^b^** |
| 1 | 0.5 | *Salvia miltiorrhiza* Bunge | 丹參 | Plantae | Lamiaceae | herbaceous | radix | decoction | anticancer**^b^** |
| 1 | 0.5 | *Momordica cochinchinensis* Spreng. | 木鱉子 | Plantae | Cucurbitaceae | liana | semen | juice | breast cancer |
| 1 | 0.5 | Bombax ceiba *L.* | 木棉根 | Plantae | Malvaceae | tree | radix | tea | lung cancer, colorectal cancer |
| 1 | 0.5 | *Akebia quinata* (Thunb. ex Houtt.) Decne. | 八月札 | Plantae | Lardizabalaceae | liana | fructus | decoction | liver cancer |
| 1 | 0.5 | *Solanum lyratum* Thunb. | 白英、白毛藤 | Plantae | Solanaceae | liana | herba | decoction | liver cancer |
| 1 | 0.5 | *Dioscorea bulbifera* L. | 黃藥子 | Plantae | Dioscoreaceae | herbaceous | rhizoma | decoction | gastric cancer |
| 1 | 0.5 | *Solanum insanum* L. | 黃水茄 | Plantae | Solanaceae | shrub | whole plant | decoction | liver cancer, skin cancer |
| 1 | 0.5 | *Chenopodium glaucum* L. | 藜麥 | Plantae | Amaranthaceae | herbaceous | herba | dish, tea | colorectal cancer |
| 1 | 0.5 | *Strobilanthes cusia* (Nees) Kuntze | 青黛 | Plantae | Acanthaceae | shrub | naturalis | crush | lung cancer, leukemia |
| 1 | 0.5 | *Gynostemma pentaphyllum* (Thunb.) Makino | 絞股藍、七葉膽 | Plantae | Cucurbitaceae | liana | herba | crush | liver cancer, skin cancer, cervical cancer |
| 1 | 0.5 | *Codonopsis pilosula* Nannf. | 黨參 | Plantae | Campanulaceae | liana | radix | decoction | anticancer**^b^** |
| 1 | 0.5 | *Xanthium strumarium* subsp. *Strumarium* | 蒼耳子 | Plantae | Asteraceae | herbaceous | fructus | decoction | anticancer**^b^** |
| 1 | 0.5 | *Sophora tonkinensis* Gagnep. | 山豆根 | Plantae | Fabaceae | shrub | radix | decoction | gastric cancer, nasopharyngeal cancer |
| 1 | 0.5 | *Broussonetia papyrifera* (L.) Vent. | 構樹 | Plantae | Moraceae | tree | folium | tea | lung cancer, liver cancer |
| 1 | 0.5 | *Psidium guajava* L. | 番石榴、芭樂 | Plantae | Myrtaceae | tree | folium | decoction | gastric cancer |
| 1 | 0.5 | *Carica papaya* L. | 番木瓜 | Plantae | Caricaceae | tree | folium | juice | oral cancer |
| 1 | 0.5 | *Ligustrum lucidum* W.T.Aiton | 女貞子 | Plantae | Oleaceae | shrub | fructus | broth | breast cancer |
| 1 | 0.5 | *Euphorbia tirucalli* L. | 綠珊瑚、鹿角草 | Plantae | Euphorbiaceae | tree | caulis | decoction | colorectal cancer |
| 1 | 0.5 | *Ranunculus ternatus* Thunb. | 猫爪草 | Plantae | Ranunculaceae | herbaceous | radix | decoction | lymphoma |
| 1 | 0.5 | *Begonia fimbristipula* Hance | 紫背天葵 | Plantae | Begoniaceae | herbaceous | cormus | decoction | lymphoma |
| 1 | 0.5 | *Schisandra henryi* C.B.Clarke | 紫金血藤 | Plantae | Schisandraceae | liana | radix | decoction | breast cancer, liver cancer, gastric cancer, skin cancer, lymphoma, leukemia, splenic cancer |
| 1 | 0.5 | *Polygonum perfoliatum* L. | 扛板歸 | Plantae | Polygonaceae | liana | herba | decoction | anticancer**^b^** |
| 1 | 0.5 | *Lygodium japonicum* (Thunb.) Sw. | 海金沙 | Plantae | Lygodiaceae | liana | spora | decoction | bladder cancer, penile cancer |
| 1 | 0.5 | *Allium cepa* L. | 洋蔥 | Plantae | Amaryllidaceae | herbaceous | bulbus | broth, dish | colorectal cancer |
| 1 | 0.5 | *Allium sativum* L. | 蒜頭 | Plantae | Amaryllidaceae | herbaceous | bulbus | dish, raw | various cancers**^c^** |
| 1 | 0.5 | *Leuzea uniflora* (L.) Holub | 漏蘆、山防風 | Plantae | Asteraceae | herbaceous | radix | decoction | colorectal cancer, liver cancer |
| 1 | 0.5 | *Podophyllum pleianthum* Hance | 八角蓮、鬼臼 | Plantae | Berberidaceae | herbaceous | radix | decoction | prostate cancer |
| 1 | 0.5 | *Patrinia villosa* Juss. | 敗醬 | Plantae | Caprifoliaceae | herbaceous | herba | decoction | anticancer**^b^** |
| 1 | 0.5 | *Rhinacanthus nasutus* (L.) Kurz | 白鶴靈芝、仙鶴草 | Plantae | Acanthaceae | shrub | whole plant | tea | liver cancer |
| 1 | 0.5 | *Arctium lappa* L. | 牛蒡根 | Plantae | Asteraceae | herbaceous | radix | decoction | anticancer**^b^** |
| 1 | 0.5 | *Platycodon grandiflorus* A.DC. | 桔梗 | Plantae | Campanulaceae | herbaceous | radix | decoction | lung cancer |
| 1 | 0.5 | *Fritillaria cirrhosa* D.Don | 川貝母 | Plantae | Liliaceae | herbaceous | bulbus | decoction | lung cancer |
| 1 | 0.5 | *Gleditsia sinensis* Lam. | 皂角刺 | Plantae | Fabaceae | tree | spina | decoction | liver cancer, gastric cancer |
| 1 | 0.5 | *Tribulus terrestris* L. | 刺蒺藜 | Plantae | Zygophyllaceae | liana | fructus | decoction | liver cancer |
| 1 | 0.5 | *Zingiber officinale* Roscoe | 薑 | Plantae | Zingiberaceae | herbaceous | rhizoma | dish | anticancer^b^ |
| 1 | 0.5 | *Cryptotympana atrata* (Fabricius) | 蟬蛻 | Animalia | Cicadidae | animal | periostracum | raw | oral cancer |
| 1 | 0.5 | *Gardenia jasminoides* J.Ellis | 梔子 | Plantae | Rubiaceae | shrub | fructus | raw | liver cancer |
| 1 | 0.5 | *Morinda citrifolia* L. | 諾麗果 | Plantae | Rubiaceae | tree | fructus | raw | anticancer^b^ |
| 1 | 0.5 | *Angelica biserrata* (R.H.Shan & Yuan) C.Q.Yuan & R.H.Shan | 獨活 | Plantae | Apiaceae | herbaceous | radix | decoction | cervical cancer |
| 1 | 0.5 | *Sparganium stoloniferum* (Buch.-Ham. ex Graebn.) Buch.-Ham. ex Juz. | 三棱 | Plantae | Typhaceae | herbaceous | rhizoma | decoction | lung cancer, colorectal cancer, liver cancer, gastric cancer, esophageal cancer |
| 1 | 0.5 | *Forsythia suspensa* (Thunb.) Vahl | 連翹 | Plantae | Oleaceae | herbaceous | fructus | decoction | lung cancer, oral cancer, thyroid cancer, nasopharyngeal cancer |
| 1 | 0.5 | *Trichosanthes kirilowii* Maxim. | 栝樓根、天花粉 | Plantae | Cucurbitaceae | liana | radix | decoction | lung cancer, colorectal cancer, breast cancer, liver cancer, gastric cancer, cervical cancer |
| 1 | 0.5 | *Potentilla indica* (Andrews) Th.Wolf | 蛇莓 | Plantae | Rosaceae | herbaceous | herba | decoction | colorectal cancer, liver cancer, gastric cancer, esophageal cancer |
| 1 | 0.5 | *Oldenlandia corymbosa* L. | 水線草 | Plantae | Rubiaceae | herbaceous | herba | tea | various cancers**^c^** |
| 1 | 0.5 | *Anemarrhena asphodeloides* Bunge | 知母 | Plantae | Asparagaceae | herbaceous | rhizoma | decoction | lung cancer |
| 1 | 0.5 | *Dioscorea polystachya* Turcz. | 山藥 | Plantae | Dioscoreaceae | herbaceous | rhizoma | decoction | colorectal cancer |
| 1 | 0.5 | *Helminthostachys zeylanica* (L.) Hook. | 倒地蜈蚣 | Plantae | Ophioglossaceae | herbaceous | radix | decoction | anticancer^b^ |
| 1 | 0.5 | Huangtcia renifolia *(L.) H.Ohashi & K.Ohashi* | 腎葉山螞蝗 | Plantae | Fabaceae | herbaceous | herba | tea | renal cancer |
| 1 | 0.5 | *Orthosiphon aristatus* (Blume) Miq. | 貓鬚草、化石草 | Plantae | Lamiaceae | herbaceous | herba | decoction | liver cancer |
| 1 | 0.5 | *Gynura bicolor* (Roxb. ex Willd.) DC. | 紅鳳菜 | Plantae | Asteraceae | herbaceous | herba | dish | anticancer^b^ |
| 1 | 0.5 | *Wolfiporia extensa* (Peck) Ginns | 茯苓 | Fungi | Polyporaceae | fungi | corporis | decoction | lung cancer |
| 1 | 0.5 | *Plantago asiatica* L. | 車前草 | Plantae | Plantaginaceae | herbaceous | herba | decoction, tea | bladder cancer |
| 1 | 0.5 | *Artemisia argyi* H.Lév. & Vaniot | 艾葉 | Plantae | Asteraceae | herbaceous | folium | decoction, tea | cervical cancer |
| 1 | 0.5 | *Stemona sessilifolia* (Miq.) Miq. | 百部 | Plantae | Stemonaceae | herbaceous | radix | decoction | cervical cancer |
| 1 | 0.5 | *Lobelia chinensis* Lour. | 半邊蓮 | Plantae | Campanulaceae | herbaceous | herba | decoction | lung cancer |
| 1 | 0.5 | *Citrus aurantium* L. | 橘紅 | Plantae | Rutaceae | tree | vetum | decoction | lung cancer |
| 1 | 0.5 | *Tussilago farfara* L. | 款冬花 | Plantae | Asteraceae | herbaceous | flos | decoction | lung cancer |
| 1 | 0.5 | *Aster tataricus* L.f. | 紫菀 | Plantae | Asteraceae | herbaceous | radix, rhizoma | decoction | lung cancer |
| 1 | 0.5 | *Citrus aurantium* L. | 陳皮 | Plantae | Rutaceae | tree | vetum | decoction | lung cancer |
| 1 | 0.5 | *Polygala tenuifolia* Willd. | 遠志 | Plantae | Polygalaceae | herbaceous | radix | decoction | lung cancer |
| 1 | 0.5 | *Arnebia euchroma* I.M.Johnst. | 紫草 | Plantae | Boraginaceae | herbaceous | radix | decoction | lung cancer |
| 1 | 0.5 | *Graptopetalum paraguayense* (N.E.Br.) E.Walther | 石蓮花 | Plantae | Crassulaceae | herbaceous | folium | juice, raw | liver cancer |
| 1 | 0.5 | *Livistona chinensis* (Jacq.) R.Br. ex Mart. | 蒲葵子 | Plantae | Arecaceae | tree | semen | broth | esophageal cancer |
| 1 | 0.5 | *Gymnema sylvestre* (Retz.) R.Br. ex Sm. | 武靴藤 | Plantae | Apocynaceae | liana | radix | decoction | anticancer^b^ |
| 1 | 0.5 | *Ampelopsis glandulosa* (Wall.) Momiy. | 山葡萄 | Plantae | Vitaceae | liana | radix | decoction | anticancer^b^ |
| 1 | 0.5 | *Setaria viridis* (L.) P.Beauv. | 狗尾草、通天草 | Plantae | Poaceae | herbaceous | herba | decoction | lung cancer, liver cancer |
| 1 | 0.5 | *Triumfetta rhomboidea* Jacq. | 黃花虱母、黃花三脚破 | Plantae | Malvaceae | herbaceous | herba | decoction | lung cancer |
| 1 | 0.5 | *Clerodendrum chinense* (Osbeck) Mabb. | 臭茉莉 | Plantae | Lamiaceae | shrub | radix | decoction | lung cancer |
| 1 | 0.5 | Solanum lasiocarpum *Dunal* | 鈕仔茄 | Plantae | Solanaceae | shrub | herba | decoction | liver cancer |
| 1 | 0.5 | *Ixeris chinensis* (Thunb. ex Thunb.) Nakai | 兔兒菜、小金英 | Plantae | Asteraceae | herbaceous | herba | decoction, tea | liver cancer |
| 1 | 0.5 | *Cheilosoria chusana* (Hook.) Ching | 石壁癀、細葉碎米蕨 | Plantae | Pteridaceae | herbaceous | herba | decoction | liver cancer |
| 1 | 0.5 | *Dichondra repens* J.R.Forst. & G.Forst. | 馬蹄金 | Plantae | Convolvulaceae | liana | herba | decoction | liver cancer |
| 1 | 0.5 | *Artemisia scoparia* Waldst. & Kit. | 茵陳 | Plantae | Asteraceae | herbaceous | herba | decoction | liver cancer |
| 1 | 0.5 | *Canarium album* (Lour.) Raeusch. ex DC. | 橄欖根 | Plantae | Burseraceae | tree | radix | decoction | liver cancer |
| 1 | 0.5 | Hellenia speciosa *(J.Koenig) S.R.Dutta* | 閉鞘薑 | Plantae | Costaceae | herbaceous | rhizoma | tea | breast cancer |
| 1 | 0.5 | *Polistes mandarinus* Saussure | 露蜂房 | Animalia | Vespidae | animal | nidum | decoction | lung cancer |
| 1 | 0.5 | *Phellodendron chinense* C.K.Schneid. | 黃蘗 | Plantae | Rutaceae | tree | cortex | decoction | lymphoma |
| 1 | 0.5 | *Dysphania ambrosioides* (L.) Mosyakin & Clemants | 臭杏、土荊芥 | Plantae | Amaranthaceae | herbaceous | herba | tea | cholangiocarcinoma |
| Use of parts, preparation methods and cancer type were sequenced in mentioned frequencies order.  ^a^ The number of informants mentioned the species.  ^b^ Informant only know the species has anticancer effects, but not sure which cancers shall be treated.  ^c^ The species was treated for various cancers. | | | | | | | | | |

| **Table S2. Compare treatment of cancer types of top ten ethnomedicines with documented researches collected.** | | | | | | |
| --- | --- | --- | --- | --- | --- | --- |
| **Rank** | **Scientific name** | **Frequency^a^** | **Percentage (%)** | **Cancer types** | | |
|  |  |  |  | **Traditionally used** | **NCBI PubMed [Reference]** | **The same** |
| **1^st^** | *Taraxacum formosanum* Kitam. | 136 | 64.8 | **liver cancer**, **breast cancer**, **lung cancer**, colorectal cancer, skin cancer, anticancer**^b^**, gastric cancer, pancreatic cancer, various cancer**^c^**, esophageal cancer, leukemia, prostate cancer, thyroid cancer, lymphoma, ovarian cancer, uterine cancer, nasopharyngeal cancer, renal cancer, bladder cancer | **breast cancer** (Lin et al., 2022a), **lung cancer** (Chien et al., 2018), cervical cancer (Lin et al., 2022b), **liver cancer** (Lin et al., 2022b) | **liver cancer, breast cancer, lung cancer** |
| **2^nd^** | *Scleromitrion diffusum* (Willd.) R.J.Wang | 125 | 59.5 | **liver cancer**, **lung cancer**, gastric cancer, **colorectal cancer**, anticancer**^b^**, various cancers**^c^**, **breast cancer**, esophageal cancer, lymphoma, cervical cancer, leukemia, oral cancer, **prostate cancer**, pancreatic cancer, **uterine cancer**, **bladder cancer**, splenic cancer, small intestine cancer | **breast cancer** (Dong et al., 2014;Yeh et al., 2014;Fang et al., 2020;Jing et al., 2020;Yang et al., 2020;Li et al., 2024), renal cancer (Wong et al., 1996;Bai et al., 2024), **liver cancer** (Yang et al., 2021), **prostate cancer** (Yang et al., 2022), ovarian cancer (Xu et al., 2021), **bladder cancer** (Pan et al., 2016), **colorectal cancer** (Zhu et al., 2023a), **uterine cancer** (Pang et al., 2016), **lung cancer** (Huang et al., 2024), nasopharyngeal cancer (Wang et al., 2020a) | **liver cancer, lung cancer, colorectal cancer, breast cancer, uterine cancer, bladder cancer** |
| **3^rd^** | *Scutellaria barbata* D.Don | 122 | 58.1 | **liver cancer**, **lung cancer**, **gastric cancer**, **breast cancer**, anticancer**^b^**, **colorectal cancer**, various cancers**^c^**, esophageal cancer, **uterine cancer**, **leukemia**, nasopharyngeal cancer, oral cancer, **prostate cancer**, pancreatic cancer, lymphoma | **liver cancer** (Chui et al., 2005;Lin et al., 2006a;b;Tang et al., 2006;Dai et al., 2008a;Dai et al., 2008b;Dai et al., 2008c;Dai et al., 2008d;Dai et al., 2008e;Dai et al., 2013;Gao et al., 2014;Kan et al., 2017;Gong et al., 2018;Li et al., 2019b;Wang et al., 2019c;Feng et al., 2021a;Xu et al., 2021;Yang et al., 2021;Su et al., 2022;Shao et al., 2023), **colorectal cancer** (Goh et al., 2005;Wei et al., 2012;Wei et al., 2013;Lin et al., 2014;Zhang et al., 2014;Jiang et al., 2015;Lin et al., 2017;Sun et al., 2017;Wei et al., 2017;Yang et al., 2017;Zhang et al., 2017a;Guo et al., 2019;Li et al., 2019a;Wang et al., 2019c;Lv et al., 2021;Qi et al., 2021;Zeng et al., 2021;Liu et al., 2022;Zhu et al., 2023a), **breast cancer** (Chui et al., 2005;Fong et al., 2008;Marconett et al., 2010;Perez et al., 2010;Klawitter et al., 2011;Yeh et al., 2014;Xue et al., 2016;Zheng et al., 2018;Wang et al., 2019c;Fang et al., 2020;Jing et al., 2020;Ma et al., 2020;Yang et al., 2020;Li et al., 2024;Ma et al., 2024;Niu et al., 2024), **lung cancer** (Yin et al., 2004;Chui et al., 2005;Kim et al., 2007b;Shiau et al., 2014;Yang et al., 2014a;Yang et al., 2014b;Gong et al., 2015;Yang et al., 2015;Chen et al., 2017;Liu et al., 2018;Wang et al., 2018;Chen et al., 2021), ovarian cancer (Powell et al., 2003;Suh et al., 2007;Li et al., 2014;Zhang et al., 2017b;Xu et al., 2021;Zhang et al., 2023a), **leukemia** (Cha et al., 2004;Chui et al., 2005;Kim et al., 2007a;Shi et al., 2016;Huang et al., 2022), **prostate cancer** (Wong et al., 2009;Marconett et al., 2010;Sheng et al., 2022;Yang et al., 2022), **uterine cancer** (Lee et al., 2004;Kim et al., 2008;Pang et al., 2016;Bao et al., 2019), bladder cancer (Pan et al., 2016;Niu et al., 2020), renal cancer (Wong et al., 1996;Bai et al., 2024), lung cancer (Kim et al., 2006;Wei et al., 2007), pancreatic cancer (Wang et al., 2019b), skin cancer (Suh et al., 2007), **gastric cancer** (Shim et al., 2016), cervical cancer (Xue et al., 2022) | **liver cancer, lung cancer, gastric cancer, breast cancer, colorectal cancer, uterine cancer, leukemia, prostate cancer** |
| **4^th^** | *Zanthoxylum ailanthoides* Siebold & Zucc. | 116 | 55.2 | **colorectal cancer**, anticancer**^b^**, **liver cancer**, **lung cancer**, gastric cancer, uterine cancer | **colorectal cancer** (Chou et al., 2011b), **lung cancer** (Cao et al., 2013), leukemia (Chou et al., 2011a), **liver cancer** (Tseng et al., 2022) | **colorectal cancer**, **liver cancer**, **lung cancer** |
| **5^th^** | *Prunella vulgaris* L. | 116 | 55.2 | **lymphoma**, **breast cancer**, **liver cancer**, **thyroid cancer**, gastric cancer, anticancer**^b^**, **lung cancer**, esophageal cancer, **leukemia**, **colorectal cancer**, cholangiocarcinoma, oral cancer, various cancers**^c^**, cervical cancer, nasopharyngeal cancer, skin cancer, **uterine cancer**, brain cancer, bone cancer | **thyroid cancer** (Yin et al., 2017;Song et al., 2021a;Yu et al., 2021;Zhang et al., 2023b;Zheng et al., 2023;Zhu et al., 2023b), **breast cancer** (Gao and Xu, 2019;Hao et al., 2020;Lim et al., 2020;Zhang et al., 2020;Bai et al., 2022;Luo et al., 2022), **lung cancer** (Feng et al., 2010a;Feng et al., 2010b;Feng et al., 2011;Wang et al., 2014;Zhu et al., 2018), **liver cancer** (Kim et al., 2012;Su et al., 2016;Song et al., 2021b;Tu et al., 2024), **lymphoma** (Zhang et al., 2006;Liu et al., 2010;Fu et al., 2012;Mao et al., 2013), **colorectal cancer** (Lin et al., 2013;Fang et al., 2017;Lei et al., 2021), **leukemia** (Zhang et al., 2009;Woo et al., 2011), **uterine cancer** (Lin et al., 2020) | **lymphoma**, **breast cancer**, **liver cancer**, **thyroid cancer**, **lung cancer**, **leukemia**, **colorectal cancer**, **uterine cancer** |
| **6^th^** | *Curcuma phaeocaulis* Valeton | 83 | 39.5 | **cervical cancer**, **liver cancer**, **gastric cancer**, anticancer**^b^**, uterine cancer, ovarian cancer, **lung cancer**, lymphoma, esophageal cancer, bladder cancer, skin cancer, **colorectal cancer**, leukemia, various cancers**^c^**, **breast cancer**, nasopharyngeal cancer, brain cancer, splenic cancer | **lung cancer** (Hou et al., 2015;Xu et al., 2018;Xu et al., 2019), **colorectal cancer** (Feng et al., 2021b), **liver cancer** (Hou et al., 2015), **cervical cancer** (Hou et al., 2015), **breast cancer** (Chen et al., 2011), **gastric cancer** (Wu et al., 2022) | **cervical cancer**, **liver cancer**, **gastric cancer**, **lung cancer**, **colorectal cancer**, **breast cancer** |
| **7^th^** | *Clinacanthus nutans* (Burm.f.) Lindau | 72 | 34.3 | **lymphoma**, anticancer**^b^**, **liver cancer**, **breast cancer**, **lung cancer**, various cancers**^c^**, **oral cancer**, **renal cancer**, **colorectal cancer**, cervical cancer, **gastric cancer**, brain cancer, ovarian cancer, **uterine cancer**, prostate cancer, thyroid cancer, esophageal cancer, leukemia, bladder cancer | **breast cancer** (Quah et al., 2017;Teoh et al., 2017;Ismail et al., 2020;Mutazah et al., 2020;Chiu et al., 2021;Nordin et al., 2021;Widjaja et al., 2021;Ismail et al., 2022;Abu Bakar et al., 2023;Syarifah et al., 2023), **uterine cancer** (Yong et al., 2013;Teoh et al., 2017;Haron et al., 2019;Nik Zainuddin et al., 2020;Chiu et al., 2021), **liver cancer** (Yong et al., 2013;Huang et al., 2015;Ng et al., 2017;Quah et al., 2017), **lung cancer** (Yong et al., 2013;Fazil et al., 2016;Ng et al., 2017), **lymphoma** (Yong et al., 2013;Ismail et al., 2022), **colorectal cancer** (Yong et al., 2013;Chiu et al., 2021), **gastric cancer** (Yong et al., 2013), nasopharyngeal cancer (Ng et al., 2017), **renal cancer** (Ye et al., 2023), skin cancer (Fong et al., 2016), **oral cancer** (Yakop et al., 2018) | **lymphoma**, **liver cancer**, **breast cancer**, **lung cancer**, **oral cancer**, **renal cancer**, **colorectal cancer**, **gastric cancer**, **uterine cancer** |
| **8^th^** | *Buthus martensii* Karsch | 64 | 30.5 | liver cancer, gastric cancer, **breast cancer**, **lung cancer**, colorectal cancer, skin cancer, anticancer**^b^**, various cancers**^c^**, esophageal cancer, prostate cancer, uterine cancer, **lymphoma**, thyroid cancer, cervical cancer, brain cancer | **lung cancer** (Mao et al., 2024), **breast cancer** (Kampo et al., 2019), **lymphoma** (Gao et al., 2009), oral cancer (Satitmanwiwat et al., 2016) | **breast cancer**, **lung cancer**, **lymphoma** |
| **9^th^** | *Gynura procumbens* (Lour.) Merr. | 37 | 17.6 | **liver cancer**, anticancer**^b^**, lung cancer, gastric cancer, various cancers**^c^**, colorectal cancer, | **liver cancer** (Nisa et al., 2012;Zhang et al., 2022), breast cancer (Hew et al., 2013;Jermnak et al., 2022) | **liver cancer** |
| **10^th^** | *Rhus chinensis* var. *roxburghii* (DC.) Rehder | 30 | 14.3 | anticancer**^b^**, lung cancer, **colorectal cancer**, gastric cancer, lymphoma, nasopharyngeal cancer, prostate cancer, oral cancer, **breast cancer**, liver cancer, renal cancer | **colorectal cancer** (Wang et al., 2019a;Wang et al., 2020b;Wang et al., 2021;Wang et al., 2022), **breast cancer** (Yu et al., 2011) | **colorectal cancer, breast cancer** |
| Cancer types were listed in mentioned frequencies order. Bold showed the same cancer types between ethnomedicines and documented researches.  ^a^ The number of informants mentioned the species.  ^b^ Informant only know the species has anticancer effects, but not sure which cancers shall be treated.  ^c^ The species was treated for various cancers. | | | | | | |

References

Abu Bakar, N., Yeo, Z.L., Hussin, F., Madhavan, P., Lim, V., Jemon, K., and Prabhakaran, P. (2023). Synergistic effects of combined cisplatin and Clinacanthus nutans extract on triple negative breast cancer cells. *J Taibah Univ Med Sci* 18**,** 1220-1236.

Bai, H., Wang, R., Li, Y., Liang, X., Zhang, J., Sun, N., and Yang, J. (2022). Network Pharmacology Analysis, Molecular Docking, and In Vitro Verification Reveal the Action Mechanism of Prunella vulgaris L. in Treating Breast Cancer. *Evid Based Complement Alternat Med* 2022**,** 5481563.

Bai, Y., Chen, R., Sun, J., and Guo, Y. (2024). Evaluation of Therapeutic Mechanism of *Hedyotis Diffusa* Willd (HDW)‒ *Scutellaria Barbata* (SB) in Clear Cell Renal Cell Carcinoma via Singlecell RNA Sequencing and Network Pharmacology. *Comb Chem High Throughput Screen* 27**,** 910-921.

Bao, X., Li, L., and Xue, X. (2019). Flavonoids from Scutellaria barbata inhibit activation of tumor-associated macrophages by blocking the Toll-like receptor 4/myeloid differentiation factor 88/nuclear factor-κB signaling pathway. *J Tradit Chin Med* 39**,** 160-165.

Cao, X.L., Xu, J., Bai, G., Zhang, H., Liu, Y., Xiang, J.F., and Tang, Y.L. (2013). Isolation of anti-tumor compounds from the stem bark of Zanthoxylum ailanthoides Sieb. & Zucc. by silica gel column and counter-current chromatography. *J Chromatogr B Analyt Technol Biomed Life Sci* 929**,** 6-10.

Cha, Y.Y., Lee, E.O., Lee, H.J., Park, Y.D., Ko, S.G., Kim, D.H., Kim, H.M., Kang, I.C., and Kim, S.H. (2004). Methylene chloride fraction of Scutellaria barbata induces apoptosis in human U937 leukemia cells via the mitochondrial signaling pathway. *Clin Chim Acta* 348**,** 41-48.

Chen, C.C., Kao, C.P., Chiu, M.M., and Wang, S.H. (2017). The anti-cancer effects and mechanisms of Scutellaria barbata D. Don on CL1-5 lung cancer cells. *Oncotarget* 8**,** 109340-109357.

Chen, W.W., Gong, K.K., Yang, L.J., Dai, J.J., Zhang, Q., Wang, F., Li, X.L., Xi, S.C., and Du, J. (2021). Scutellariabarbata D. Don extraction selectively targets stemness-prone NSCLC cells by attenuating SOX2/SMO/GLI1 network loop. *J Ethnopharmacol* 265**,** 113295.

Chen, X., Pei, L., Zhong, Z., Guo, J., Zhang, Q., and Wang, Y. (2011). Anti-tumor potential of ethanol extract of Curcuma phaeocaulis Valeton against breast cancer cells. *Phytomedicine* 18**,** 1238-1243.

Chien, J.T., Chang, R.H., Hsieh, C.H., Hsu, C.Y., and Wang, C.C. (2018). Antioxidant property of Taraxacum formosanum Kitam and its antitumor activity in non-small-cell lung cancer cells. *Phytomedicine* 49**,** 1-10.

Chiu, H.I., Che Mood, C.N.A., Mohamad Zain, N.N., Ramachandran, M.R., Yahaya, N., Nik Mohamed Kamal, N.N.S., Tung, W.H., Yong, Y.K., Lee, C.K., and Lim, V. (2021). Biogenic Silver Nanoparticles of Clinacanthus nutans as Antioxidant with Antimicrobial and Cytotoxic Effects. *Bioinorg Chem Appl* 2021**,** 9920890.

Chou, S.T., Chan, H.H., Peng, H.Y., Liou, M.J., and Wu, T.S. (2011a). Isolation of substances with antiproliferative and apoptosis-inducing activities against leukemia cells from the leaves of Zanthoxylum ailanthoides Sieb. & Zucc. *Phytomedicine* 18**,** 344-348.

Chou, S.T., Peng, H.Y., Chang, C.T., Yang, J.S., Chung, H.K., Yang, S.T., Wood, W.G., and Chung, J.G. (2011b). Zanthoxylum ailanthoides Sieb and Zucc. extract inhibits growth and induces cell death through G2/M-phase arrest and activation of apoptotic signals in colo 205 human colon adenocarcinoma cells. *Anticancer Res* 31**,** 1667-1676.

Chui, C.H., Lau, F.Y., Tang, J.C., Kan, K.L., Cheng, G.Y., Wong, R.S., Kok, S.H., Lai, P.B., Ho, R., Gambari, R., and Chan, A.S. (2005). Activities of fresh juice of Scutellaria barbata and warmed water extract of Radix Sophorae Tonkinensis on anti-proliferation and apoptosis of human cancer cell lines. *Int J Mol Med* 16**,** 337-341.

Dai, Z., Liu, X., Ji, Z., Liu, L., Kang, H., Wang, X., and Diao, Y. (2008a). The effect-enhancing and toxicity-reducing action of the extract of herba Scutellariae barbatae for chemotherapy in hepatoma H22 tumor-bearing mice. *J Tradit Chin Med* 28**,** 205-210.

Dai, Z.J., Liu, X.X., Xue, Q., Ji, Z.Z., Wang, X.J., Kang, H.F., Guan, H.T., Ma, X.B., and Ren, H.T. (2008b). Anti-proliferative and apoptosis-inducing activity of Scutellaria barbate containing serum on mouse's hepatoma H22 cells. *Zhong Yao Cai* 31**,** 550-553.

Dai, Z.J., Wang, B.F., Lu, W.F., Wang, Z.D., Ma, X.B., Min, W.L., Kang, H.F., Wang, X.J., and Wu, W.Y. (2013). Total flavonoids of Scutellaria barbata inhibit invasion of hepatocarcinoma via MMP/TIMP in vitro. *Molecules* 18**,** 934-950.

Dai, Z.J., Wang, X.J., Ji, Z.Z., Li, Z.F., Tang, W., Kang, H.F., Ma, X.B., and Liu, L. (2008c). Scutellaria barbata extract enhances efficacy and reduces toxicity of chemotherapy in hepatoma H22-bearing mice. *Zhong Xi Yi Jie He Xue Bao* 6**,** 720-724.

Dai, Z.J., Wang, X.J., Li, Z.F., Ji, Z.Z., Ren, H.T., Tang, W., Liu, X.X., Kang, H.F., Guan, H.T., and Song, L.Q. (2008d). Scutellaria barbate extract induces apoptosis of hepatoma H22 cells via the mitochondrial pathway involving caspase-3. *World J Gastroenterol* 14**,** 7321-7328.

Dai, Z.J., Wang, X.J., Xue, Q., Ji, Z.Z., Liu, X.X., Kang, H.F., Guan, H.T., Ma, X.B., and Ren, H.T. (2008e). Effects of Scutellaria Barbata drug-containing serum on apoptosis and mitochondrial transmembrane potential of hepatoma H22 cells. *Zhong Xi Yi Jie He Xue Bao* 6**,** 821-826.

Dong, Q., Ling, B., Gao, B., Maley, J., Sammynaiken, R., and Yang, J. (2014). Hedyotis diffusa water extract diminished the cytotoxic effects of chemotherapy drugs against human breast cancer MCF7 cells. *Nat Prod Commun* 9**,** 699-700.

Fang, T., Yan, Y.X., Yang, Y., Lv, Y.X., Chang, Q.Q., and Zhang, D.D. (2020). Ethyl Acetate Fraction from *Hedyotis diffusa* plus *Scutellaria barbata* Suppresses Migration of Bone-Metastatic Breast Cancer Cells via OPN-FAK/ERK/NF-κB Axis. *Evid Based Complement Alternat Med* 2020**,** 3573240.

Fang, Y., Zhang, L., Feng, J., Lin, W., Cai, Q., and Peng, J. (2017). Spica Prunellae extract suppresses the growth of human colon carcinoma cells by targeting multiple oncogenes via activating miR-34a. *Oncol Rep* 38**,** 1895-1901.

Fazil, F.N., Azzimi, N.S., Yahaya, B.H., Kamalaldin, N.A., and Zubairi, S.I. (2016). Kinetics Extraction Modelling and Antiproliferative Activity of Clinacanthus nutans Water Extract. *ScientificWorldJournal* 2016**,** 7370536.

Feng, L., Au-Yeung, W., Xu, Y.H., Wang, S.S., Zhu, Q., and Xiang, P. (2011). Oleanolic acid from Prunella Vulgaris L. induces SPC-A-1 cell line apoptosis via regulation of Bax, Bad and Bcl-2 expression. *Asian Pac J Cancer Prev* 12**,** 403-408.

Feng, L., Jia, X., Zhu, M., Chen, Y., and Shi, F. (2010a). Chemoprevention by Prunella vulgaris L. extract of non-small cell lung cancer via promoting apoptosis and regulating the cell cycle. *Asian Pac J Cancer Prev* 11**,** 1355-1358.

Feng, L., Jia, X.B., Jiang, J., Zhu, M.M., Chen, Y., Tan, X.B., and Shi, F. (2010b). Combination of active components enhances the efficacy of Prunella in prevention and treatment of lung cancer. *Molecules* 15**,** 7893-7906.

Feng, P.P., Qi, Y.K., Li, N., and Fei, H.R. (2021a). Scutebarbatine A induces cytotoxicity in hepatocellular carcinoma via activation of the MAPK and ER stress signaling pathways. *J Biochem Mol Toxicol* 35**,** e22731.

Feng, Y., Deng, L., Guo, H., Zhao, Y., Peng, F., Wang, G., and Yu, C. (2021b). The Anti-Colon Cancer Effects of Essential Oil of Curcuma phaeocaulis Through Tumour Vessel Normalisation. *Front Oncol* 11**,** 728464.

Fong, S., Shoemaker, M., Cadaoas, J., Lo, A., Liao, W., Tagliaferri, M., Cohen, I., and Shtivelman, E. (2008). Molecular mechanisms underlying selective cytotoxic activity of BZL101, an extract of Scutellaria barbata, towards breast cancer cells. *Cancer Biol Ther* 7**,** 577-586.

Fong, S.Y., Piva, T., Dekiwadia, C., Urban, S., and Huynh, T. (2016). Comparison of cytotoxicity between extracts of Clinacanthus nutans (Burm. f.) Lindau leaves from different locations and the induction of apoptosis by the crude methanol leaf extract in D24 human melanoma cells. *BMC Complement Altern Med* 16**,** 368.

Fu, X.R., Sun, Z.C., and Zhang, M.Z. (2012). [Experimental study of extract from Prunella vulgaris inducing B, T lymphoma cell apoptosis]. *Zhong Yao Cai* 35**,** 433-438.

Gao, F., Li, H., Chen, Y.D., Yu, X.N., Wang, R., and Chen, X.L. (2009). Upregulation of PTEN involved in scorpion venom-induced apoptosis in a lymphoma cell line. *Leuk Lymphoma* 50**,** 633-641.

Gao, J., Lu, W.F., Dai, Z.J., Lin, S., Zhao, Y., Li, S., Zhao, N.N., Wang, X.J., Kang, H.F., Ma, X.B., and Zhang, W.G. (2014). Induction of apoptosis by total flavonoids from Scutellaria barbata D. Don in human hepatocarcinoma MHCC97-H cells via the mitochondrial pathway. *Tumour Biol* 35**,** 2549-2559.

Gao, W., and Xu, H. (2019). Root extract of Prunella vulgaris inhibits in vitro and in vivo carcinogenesis in MCF-5 human breast carcinoma via suppression of angiogenesis, induction of apoptosis, cell cycle arrest and modulation of PI3K/AKT signalling pathway. *J buon* 24**,** 549-554.

Goh, D., Lee, Y.H., and Ong, E.S. (2005). Inhibitory effects of a chemically standardized extract from Scutellaria barbata in human colon cancer cell lines, LoVo. *J Agric Food Chem* 53**,** 8197-8204.

Gong, B., Kao, Y., Zhang, C., Sun, F., and Zhao, H. (2018). Systematic Investigation of Scutellariae Barbatae Herba for Treating Hepatocellular Carcinoma Based on Network Pharmacology. *Evid Based Complement Alternat Med* 2018**,** 4365739.

Gong, T., Wang, C.F., Yuan, J.R., Li, Y., Gu, J.F., Zhao, B.J., Zhang, L., Jia, X.B., Feng, L., and Liu, S.L. (2015). Inhibition of Tumor Growth and Immunomodulatory Effects of Flavonoids and Scutebarbatines of Scutellaria barbata D. Don in Lewis-Bearing C57BL/6 Mice. *Evid Based Complement Alternat Med* 2015**,** 630760.

Guo, F., Yang, F., and Zhu, Y.H. (2019). Scutellarein from Scutellaria barbata induces apoptosis of human colon cancer HCT116 cells through the ROS-mediated mitochondria-dependent pathway. *Nat Prod Res* 33**,** 2372-2375.

Hao, J., Ding, X.L., Yang, X., and Wu, X.Z. (2020). Prunella vulgaris Polysaccharide Inhibits Growth and Migration of Breast Carcinoma-Associated Fibroblasts by Suppressing Expression of Basic Fibroblast Growth Factor. *Chin J Integr Med* 26**,** 270-276.

Haron, N.H., Md Toha, Z., Abas, R., Hamdan, M.R., Azman, N., Khairuddean, M., and Arsad, H. (2019). In Vitro Cytotoxic Activity of Clinacanthus nutans Leaf Extracts Against HeLa Cells. *Asian Pac J Cancer Prev* 20**,** 601-609.

Hew, C.S., Khoo, B.Y., and Gam, L.H. (2013). The anti-cancer property of proteins extracted from Gynura procumbens (Lour.) Merr. *PLoS One* 8**,** e68524.

Hou, Y., Lu, C.L., Zeng, Q.H., and Jiang, J.G. (2015). Anti-inflammatory, antioxidant and antitumor activities of ingredients of Curcuma phaeocaulis Val. *Excli j* 14**,** 706-713.

Huang, D., Guo, W., Gao, J., Chen, J., and Olatunji, J.O. (2015). Clinacanthus nutans (Burm. f.) Lindau Ethanol Extract Inhibits Hepatoma in Mice through Upregulation of the Immune Response. *Molecules* 20**,** 17405-17428.

Huang, S., Wu, M., Deng, M., Yang, S., Wang, X., and Wang, Z. (2024). Exploring the mechanism of Scleromitrion diffusum (Willd.) in treating lung cancer based on network pharmacology and experimental validation. *Nat Prod Res***,** 1-5.

Huang, Z., Yang, Y., Fan, X., and Ma, W. (2022). Network pharmacology-based investigation and experimental validation of the mechanism of scutellarin in the treatment of acute myeloid leukemia. *Front Pharmacol* 13**,** 952677.

Ismail, N.Z., Md Saad, S., Adebayo, I.A., Md Toha, Z., Abas, R., Mohamad Zain, N.N., and Arsad, H. (2022). The antiproliferative and apoptotic potential of Clinacanthus nutans against human breast cancer cells through targeted apoptosis pathway. *Environ Sci Pollut Res Int* 29**,** 81685-81702.

Ismail, N.Z., Md Toha, Z., Muhamad, M., Nik Mohamed Kamal, N.N.S., Mohamad Zain, N.N., and Arsad, H. (2020). Antioxidant Effects, Antiproliferative Effects, and Molecular Docking of Clinacanthus nutans Leaf Extracts. *Molecules* 25.

Jermnak, U., Supsavhad, W., Kunakornsawat, S., Jaroensong, T., Watcharasit, P., Visitnonthachai, D., Pairor, S., and Phaochoosak, N. (2022). Anti-cancer potentials of Gynura procumbens leaves extract against two canine mammary cancer cell lines. *Vet Med Sci* 8**,** 69-84.

Jiang, Q., Li, Q., Chen, H., Shen, A., Cai, Q., Lin, J., and Peng, J. (2015). Scutellaria barbata D. Don inhibits growth and induces apoptosis by suppressing IL-6-inducible STAT3 pathway activation in human colorectal cancer cells. *Exp Ther Med* 10**,** 1602-1608.

Jing, X.U., Feng-Qin, S., Ke-Xin, D.U., Jia, D., and Dong-Yun, L.I. (2020). Mechanism of "*Scutellaria barbata*-*Hedyotis diffusa*" against breast cancer based on network pharmacology. *Zhongguo Zhong Yao Za Zhi* 45**,** 4448-4454.

Kampo, S., Ahmmed, B., Zhou, T., Owusu, L., Anabah, T.W., Doudou, N.R., Kuugbee, E.D., Cui, Y., Lu, Z., Yan, Q., and Wen, Q.P. (2019). Scorpion Venom Analgesic Peptide, BmK AGAP Inhibits Stemness, and Epithelial-Mesenchymal Transition by Down-Regulating PTX3 in Breast Cancer. *Front Oncol* 9**,** 21.

Kan, X., Zhang, W., You, R., Niu, Y., Guo, J., and Xue, J. (2017). Scutellaria barbata D. Don extract inhibits the tumor growth through down-regulating of Treg cells and manipulating Th1/Th17 immune response in hepatoma H22-bearing mice. *BMC Complement Altern Med* 17**,** 41.

Kim, E.K., Kwon, K.B., Han, M.J., Song, M.Y., Lee, J.H., Ko, Y.S., Shin, B.C., Yu, J., Lee, Y.R., Ryu, D.G., Park, J.W., and Park, B.H. (2007a). Induction of G1 arrest and apoptosis by Scutellaria barbata in the human promyelocytic leukemia HL-60 cell line. *Int J Mol Med* 20**,** 123-128.

Kim, J.H., Lee, E.O., Lee, H.J., Ku, J.S., Lee, M.H., Yang, D.C., and Kim, S.H. (2006). Caspase activation and extracellular signal-regulated kinase/Akt inhibition were involved in luteolin-induced apoptosis in Lewis lung carcinoma cells. *Ann N Y Acad Sci* 1090**,** 147-160.

Kim, J.H., Lee, E.O., Lee, H.J., Ku, J.S., Lee, M.H., Yang, D.C., and Kim, S.H. (2007b). Caspase activation and extracellular signal-regulated kinase/Akt inhibition were involved in luteolin-induced apoptosis in Lewis lung carcinoma cells. *Ann N Y Acad Sci* 1095**,** 598-611.

Kim, K.W., Jin, U.H., Kim, D.I., Lee, T.K., Kim, M.S., Oh, M.J., Kim, M.S., Kwon, D.Y., Lee, Y.C., and Kim, C.H. (2008). Antiproliferative effect of Scutellaria barbata D. Don. on cultured human uterine leiomyoma cells by down-regulation of the expression of Bcl-2 protein. *Phytother Res* 22**,** 583-590.

Kim, S.H., Huang, C.Y., Tsai, C.Y., Lu, S.Y., Chiu, C.C., and Fang, K. (2012). The aqueous extract of Prunella vulgaris suppresses cell invasion and migration in human liver cancer cells by attenuating matrix metalloproteinases. *Am J Chin Med* 40**,** 643-656.

Klawitter, J., Klawitter, J., Gurshtein, J., Corby, K., Fong, S., Tagliaferri, M., Quattrochi, L., Cohen, I., Shtivelman, E., and Christians, U. (2011). Bezielle (BZL101)-induced oxidative stress damage followed by redistribution of metabolic fluxes in breast cancer cells: a combined proteomic and metabolomic study. *Int J Cancer* 129**,** 2945-2957.

Lee, T.K., Cho, H.L., Kim, D.I., Lee, Y.C., and Kim, C.H. (2004). Scutellaria barbata D. Don induces c-fos gene expression in human uterine leiomyomal cells by activating beta2-adrenergic receptors. *Int J Gynecol Cancer* 14**,** 526-531.

Lei, Y., Yuan, H., Gai, L., Wu, X., and Luo, Z. (2021). Uncovering Active Ingredients and Mechanisms of Spica Prunellae in the Treatment of Colon Adenocarcinoma: A Study Based on Network Pharmacology and Bioinformatics. *Comb Chem High Throughput Screen* 24**,** 306-318.

Li, H., Su, J., Jiang, J., Li, Y., Gan, Z., Ding, Y., Li, Y., Liu, J., Wang, S., and Ke, Y. (2019a). Characterization of polysaccharide from Scutellaria barbata and its antagonistic effect on the migration and invasion of HT-29 colorectal cancer cells induced by TGF-β1. *Int J Biol Macromol* 131**,** 886-895.

Li, J., Wang, Y., Lei, J.C., Hao, Y., Yang, Y., Yang, C.X., and Yu, J.Q. (2014). Sensitisation of ovarian cancer cells to cisplatin by flavonoids from Scutellaria barbata. *Nat Prod Res* 28**,** 683-689.

Li, L., Xu, X., Wu, L., Zhu, H., He, Z., Zhang, B., Chi, Y., and Song, G. (2019b). Scutellaria barbata polysaccharides inhibit tumor growth and affect the serum proteomic profiling of hepatoma H22‑bearing mice. *Mol Med Rep* 19**,** 2254-2262.

Li, Z., Li, J., Liu, X., Sun, Z., and Sun, X. (2024). Ethyl Acetate Fraction from Hedyotis Diffusa Plus *Scutellaria Barbata* Inhibits the Progression of Breast Cancer via Targeting LMO1 and AKT/Mtor Signaling Pathway. *Comb Chem High Throughput Screen* 27**,** 1735-1744.

Lim, G.E., Sung, J.Y., Yu, S., Kim, Y., Shim, J., Kim, H.J., Cho, M.L., Lee, J.S., and Kim, Y.N. (2020). Pygenic Acid A (PA) Sensitizes Metastatic Breast Cancer Cells to Anoikis and Inhibits Metastasis In Vivo. *Int J Mol Sci* 21.

Lin, C.J., Chen, J.T., Yeh, L.J., Yang, R.C., Huang, S.M., and Chen, T.W. (2022a). Characteristics of the Cytotoxicity of Taraxacum mongolicum and Taraxacum formosanum in Human Breast Cancer Cells. *Int J Mol Sci* 23.

Lin, C.J., Liu, S.T., Yang, R.C., Wang, L.H., Tsai, C.C., Chen, T.W., and Huang, S.M. (2022b). Anticancer Effects of Taraxacum via Cell Cycle Arrest, Necrosis, Apoptosis, and Endoplasmic Reticulum Stress. *Am J Chin Med* 50**,** 569-587.

Lin, J., Chen, Y., Cai, Q., Wei, L., Zhan, Y., Shen, A., Sferra, T.J., and Peng, J. (2014). Scutellaria Barbata D Don Inhibits Colorectal Cancer Growth via Suppression of Multiple Signaling Pathways. *Integr Cancer Ther* 13**,** 240-248.

Lin, J., Feng, J., Yang, H., Yan, Z., Li, Q., Wei, L., Lai, Z., Jin, Y., and Peng, J. (2017). Scutellaria barbata D. Don inhibits 5-fluorouracil resistance in colorectal cancer by regulating PI3K/AKT pathway. *Oncol Rep* 38**,** 2293-2300.

Lin, J.M., Liu, Y., and Luo, R.C. (2006a). Effect of Scutellaria barbata extract against human hepatocellular Hep-G2 cell proliferation and its mechanism. *Nan Fang Yi Ke Da Xue Xue Bao* 26**,** 975-977.

Lin, J.M., Liu, Y., and Luo, R.C. (2006b). [Inhibition activity of Scutellariae barbata extracts against human hepatocellular carcinoma cells]. *Nan Fang Yi Ke Da Xue Xue Bao* 26**,** 591-593.

Lin, W., Zheng, L., Zhuang, Q., Zhao, J., Cao, Z., Zeng, J., Lin, S., Xu, W., and Peng, J. (2013). Spica prunellae promotes cancer cell apoptosis, inhibits cell proliferation and tumor angiogenesis in a mouse model of colorectal cancer via suppression of stat3 pathway. *BMC Complement Altern Med* 13**,** 144.

Lin, Y., Yang, C., Tang, J., Li, C., Zhang, Z.M., Xia, B.H., Li, Y.M., He, Q.Z., Lin, L.M., and Liao, D.F. (2020). Characterization and anti-uterine tumor effect of extract from Prunella vulgaris L. *BMC Complement Med Ther* 20**,** 189.

Liu, J., Jiang, M., Li, Z., Zhang, X., Li, X., Hao, Y., Su, X., Zhu, J., Zheng, C., Xiao, W., and Wang, Y. (2018). A Novel Systems Pharmacology Method to Investigate Molecular Mechanisms of Scutellaria barbata D. Don for Non-small Cell Lung Cancer. *Front Pharmacol* 9**,** 1473.

Liu, L., Liu, T., Tao, W., Liao, N., Yan, Q., Li, L., Tan, J., Shen, W., Cheng, H., and Sun, D. (2022). Flavonoids from *Scutellaria barbata* D. Don exert antitumor activity in colorectal cancer through inhibited autophagy and promoted apoptosis via ATF4/sestrin2 pathway. *Phytomedicine* 99**,** 154007.

Liu, X.K., Wang, L., and Zhang, M.Z. (2010). [Involvement of JNK and caspase-3 in human lymphoma cell apoptosis induced by Prunella vulgaris]. *Zhonghua Yi Xue Za Zhi* 90**,** 690-693.

Luo, H., Zhao, L., Li, Y., Xia, B., Lin, Y., Xie, J., Wu, P., Liao, D., Zhang, Z., and Lin, L. (2022). An in vivo and in vitro assessment of the anti-breast cancer activity of crude extract and fractions from Prunella vulgaris L. *Heliyon* 8**,** e11183.

Lv, Y.X., Pan, H.R., Song, X.Y., Chang, Q.Q., and Zhang, D.D. (2021). *Hedyotis diffusa* plus *Scutellaria barbata* Suppress the Growth of Non-Small-Cell Lung Cancer via NLRP3/NF-κB/MAPK Signaling Pathways. *Evid Based Complement Alternat Med* 2021**,** 6666499.

Ma, H., Yue, G.G., Lee, J.K., Gao, S., Yuen, K.K., Cheng, W., Li, X., and Lau, C.B. (2024). Scutellarin, a flavonoid compound from *Scutellaria barbata*, suppresses growth of breast cancer stem cells in vitro and in tumor-bearing mice. *Phytomedicine* 128**,** 155418.

Ma, T.T., Zhang, G.L., Dai, C.F., Zhang, B.R., Cao, K.X., Wang, C.G., Yang, G.W., and Wang, X.M. (2020). *Scutellaria barbata* and *Hedyotis diffusa* herb pair for breast cancer treatment: Potential mechanism based on network pharmacology. *J Ethnopharmacol* 259**,** 112929.

Mao, Q.Y., Wang, X.Q., Lin, F., Yu, M.W., Fan, H.T., Zheng, Q., Liu, L.C., Zhang, C.C., Li, D.R., and Lin, H.S. (2024). Scorpiones, Scolopendra and Gekko Inhibit Lung Cancer Growth and Metastasis by Ameliorating Hypoxic Tumor Microenvironment via PI3K/AKT/mTOR/HIF-1α Signaling Pathway. *Chin J Integr Med* 30**,** 799-808.

Mao, X., Wang, G., Zhang, W., and Li, S. (2013). A study on inhibitory effect of Spica prunellae extract on T lymphoma cell EL-4 tumour. *Afr J Tradit Complement Altern Med* 10**,** 318-324.

Marconett, C.N., Morgenstern, T.J., San Roman, A.K., Sundar, S.N., Singhal, A.K., and Firestone, G.L. (2010). BZL101, a phytochemical extract from the Scutellaria barbata plant, disrupts proliferation of human breast and prostate cancer cells through distinct mechanisms dependent on the cancer cell phenotype. *Cancer Biol Ther* 10**,** 397-405.

Mutazah, R., Hamid, H.A., Mazila Ramli, A.N., Fasihi Mohd Aluwi, M.F., and Yusoff, M.M. (2020). In vitro cytotoxicity of Clinacanthus nutans fractions on breast cancer cells and molecular docking study of sulphur containing compounds against caspase-3. *Food Chem Toxicol* 135**,** 110869.

Ng, P.Y., Chye, S.M., Ng Ch, H., Koh, R.Y., Tiong, Y.L., Pui, L.P., Tan, Y.H., Lim, C.S., and Ng Kh, Y. (2017). Clinacanthus Nutans Hexane Extracts Induce Apoptosis Through a Caspase-Dependent Pathway in Human Cancer Cell Lines. *Asian Pac J Cancer Prev* 18**,** 917-926.

Nik Zainuddin, N.a.S., Muhammad, H., Nik Hassan, N.F., Othman, N.H., and Zakaria, Y. (2020). Clinacanthus nutans Standardized Fraction Arrested SiHa Cells at G1/S and Induced Apoptosis via Upregulation of p53. *J Pharm Bioallied Sci* 12**,** S768-s776.

Nisa, F., Hermawan, A., Murwanti, R., and Meiyanto, E. (2012). Antiproliferative effect of gynura procumbens (lour.) Merr. Leaves etanolic extract on 7,12-dimethylbenz(a)antracene induced male rat liver. *Adv Pharm Bull* 2**,** 99-106.

Niu, J., Hu, J., and Wang, Z. (2024). *Scutellaria barbata* D.Don extract regulates Ezrin-mediated triple negative breast cancer progress via suppressing the RhoA /ROCK1 signaling. *Toxicol Res (Camb)* 13**,** tfae033.

Niu, W., Xu, L., Li, J., Zhai, Y., Sun, Z., Shi, W., Jiang, Y., Ma, C., Lin, H., Guo, Y., and Liu, Z. (2020). Polyphyllin II inhibits human bladder cancer migration and invasion by regulating EMT-associated factors and MMPs. *Oncol Lett* 20**,** 2928-2936.

Nordin, F.J., Pearanpan, L., Chan, K.M., Kumolosasi, E., Yong, Y.K., Shaari, K., and Rajab, N.F. (2021). Immunomodulatory potential of Clinacanthus nutans extracts in the co-culture of triple-negative breast cancer cells, MDA-MB-231, and THP-1 macrophages. *PLoS One* 16**,** e0256012.

Pan, L.T., Sheung, Y., Guo, W.P., Rong, Z.B., and Cai, Z.M. (2016). Hedyotis diffusa plus Scutellaria barbata Induce Bladder Cancer Cell Apoptosis by Inhibiting Akt Signaling Pathway through Downregulating miR-155 Expression. *Evid Based Complement Alternat Med* 2016**,** 9174903.

Pang, Y.N., Guo, J., Yan, H., Rong, R., Gong, L.L., Jiang, H.Q., and Lv, Q.T. (2016). The Component Analysis of Petroleum Ether Extract in *Scutellariae barbatae*, *Hedyotis diffusa* and the Herb Pair and the Investigation of Anti-Endometrial Cancer Cells Activity. *Zhong Yao Cai* 39**,** 789-794.

Perez, A.T., Arun, B., Tripathy, D., Tagliaferri, M.A., Shaw, H.S., Kimmick, G.G., Cohen, I., Shtivelman, E., Caygill, K.A., Grady, D., Schactman, M., and Shapiro, C.L. (2010). A phase 1B dose escalation trial of Scutellaria barbata (BZL101) for patients with metastatic breast cancer. *Breast Cancer Res Treat* 120**,** 111-118.

Powell, C.B., Fung, P., Jackson, J., Dall'era, J., Lewkowicz, D., Cohen, I., and Smith-Mccune, K. (2003). Aqueous extract of herba Scutellaria barbatae, a chinese herb used for ovarian cancer, induces apoptosis of ovarian cancer cell lines. *Gynecol Oncol* 91**,** 332-340.

Qi, X., Xu, H., Zhang, P., Chen, G., Chen, Z., Fang, C., and Lin, L. (2021). Investigating the Mechanism of Scutellariae barbata Herba in the Treatment of Colorectal Cancer by Network Pharmacology and Molecular Docking. *Evid Based Complement Alternat Med* 2021**,** 3905367.

Quah, S.Y., Chin, J.H., Akowuah, G.A., Khalivulla, S.I., Yeong, S.W., and Sabu, M.C. (2017). Cytotoxicity and cytochrome P450 inhibitory activities of Clinacanthus nutans. *Drug Metab Pers Ther* 32**,** 59-65.

Satitmanwiwat, S., Changsangfa, C., Khanuengthong, A., Promthep, K., Roytrakul, S., Arpornsuwan, T., Saikhun, K., and Sritanaudomchai, H. (2016). The scorpion venom peptide BmKn2 induces apoptosis in cancerous but not in normal human oral cells. *Biomed Pharmacother* 84**,** 1042-1050.

Shao, H., Chen, J., Li, A., Ma, L., Tang, Y., Chen, H., Chen, Y., and Liu, J. (2023). Salvigenin Suppresses Hepatocellular Carcinoma Glycolysis and Chemoresistance Through Inactivating the PI3K/AKT/GSK-3β Pathway. *Appl Biochem Biotechnol* 195**,** 5217-5237.

Sheng, D., Zhao, B., Zhu, W., Wang, T., and Peng, Y. (2022). *Scutellaria barbata* D.Don (SBD) extracts suppressed tumor growth, metastasis and angiogenesis in Prostate cancer via PI3K/Akt pathway. *BMC Complement Med Ther* 22**,** 120.

Shi, R., Guo, S.Q., Liu, S., Li, Z.S., and Li, J.J. (2016). [Effects of Scutellaria Barbata on VEGF Expression in K562 Cells]. *Zhongguo Shi Yan Xue Ye Xue Za Zhi* 24**,** 1339-1342.

Shiau, A.L., Shen, Y.T., Hsieh, J.L., Wu, C.L., and Lee, C.H. (2014). Scutellaria barbata inhibits angiogenesis through downregulation of HIF-1 α in lung tumor. *Environ Toxicol* 29**,** 363-370.

Shim, J.H., Gim, H., Lee, S., and Kim, B.J. (2016). Inductions of Caspase-, MAPK- and ROS-dependent Apoptosis and Chemotherapeutic Effects Caused by an Ethanol Extract of Scutellaria barbata D. Don in Human Gastric Adenocarcinom Cells. *J Pharmacopuncture* 19**,** 129-136.

Song, J., Zhang, Z., Hu, Y., Li, Z., Wan, Y., Liu, J., Chu, X., Wei, Q., Zhao, M., and Yang, X. (2021a). An aqueous extract of Prunella vulgaris L. inhibits the growth of papillary thyroid carcinoma by inducing autophagy in vivo and in vitro. *Phytother Res* 35**,** 2691-2702.

Song, Y.G., Kang, L., Tian, S., Cui, L.L., Li, Y., Bai, M., Fang, X.Y., Cao, L.H., Coleman, K., and Miao, M.S. (2021b). Study on the anti-hepatocarcinoma effect and molecular mechanism of Prunella vulgaris total flavonoids. *J Ethnopharmacol* 273**,** 113891.

Su, W., Wu, L., Liang, Q., Lin, X., Xu, X., Yu, S., Lin, Y., Zhou, J., Fu, Y., Gao, X., Zhang, B., Li, L., Li, D., Yin, Y., and Song, G. (2022). Extraction Optimization, Structural Characterization, and Anti-Hepatoma Activity of Acidic Polysaccharides From *Scutellaria barbata* D. Don. *Front Pharmacol* 13**,** 827782.

Su, Y.C., Lin, I.H., Siao, Y.M., Liu, C.J., and Yeh, C.C. (2016). Modulation of the Tumor Metastatic Microenvironment and Multiple Signal Pathways by Prunella vulgaris in Human Hepatocellular Carcinoma. *Am J Chin Med* 44**,** 835-849.

Suh, S.J., Yoon, J.W., Lee, T.K., Jin, U.H., Kim, S.L., Kim, M.S., Kwon, D.Y., Lee, Y.C., and Kim, C.H. (2007). Chemoprevention of Scutellaria bardata on human cancer cells and tumorigenesis in skin cancer. *Phytother Res* 21**,** 135-141.

Sun, P., Sun, D., and Wang, X. (2017). Effects of Scutellaria barbata polysaccharide on the proliferation, apoptosis and EMT of human colon cancer HT29 Cells. *Carbohydr Polym* 167**,** 90-96.

Syarifah, S., Rambe, A.S., Putra, A., Ichwan, M., Pane, Y.S., Muhar, A.M., Khatib, A., Munir, D., Rusda, M., and Amin, M.M. (2023). Water Extract Have Superior Cytotoxic Effect Than Ethanolic Extract of Clinacanthus Nutans Leaves in Breast Cancer Stem Cells. *Acta Inform Med* 32**,** 4-10.

Tang, P.M., Chan, J.Y., Au, S.W., Kong, S.K., Tsui, S.K., Waye, M.M., Mak, T.C., Fong, W.P., and Fung, K.P. (2006). Pheophorbide a, an active compound isolated from Scutellaria barbata, possesses photodynamic activities by inducing apoptosis in human hepatocellular carcinoma. *Cancer Biol Ther* 5**,** 1111-1116.

Teoh, P.L., Cheng, A.Y., Liau, M., Lem, F.F., Kaling, G.P., Chua, F.N., and Cheong, B.E. (2017). Chemical composition and cytotoxic properties of Clinacanthus nutans root extracts. *Pharm Biol* 55**,** 394-401.

Tseng, T.H., Wang, C.J., Lee, Y.J., Shao, Y.C., Shen, C.H., Lee, K.C., Tung, S.Y., and Kuo, H.C. (2022). Suppression of the Proliferation of Huh7 Hepatoma Cells Involving the Downregulation of Mutant p53 Protein and Inactivation of the STAT 3 Pathway with Ailanthoidol. *Int J Mol Sci* 23.

Tu, H., Feng, Y., Wang, W., Zhou, H., Cai, Q., and Feng, Y. (2024). Exploring the mechanism of bioactive components of Prunella vulgaris L. in treating hepatocellular carcinoma based on network pharmacology. *Chem Biol Drug Des* 103**,** e14413.

Wang, C.Y., Wang, T.C., Liang, W.M., Hung, C.H., Chiou, J.S., Chen, C.J., Tsai, F.J., Huang, S.T., Chang, T.Y., Lin, T.H., Liao, C.C., Huang, S.M., Li, T.M., and Lin, Y.J. (2020a). Effect of Chinese Herbal Medicine Therapy on Overall and Cancer Related Mortality in Patients With Advanced Nasopharyngeal Carcinoma in Taiwan. *Front Pharmacol* 11**,** 607413.

Wang, G., Wang, Y.Z., Yu, Y., and Wang, J.J. (2019a). Inhibitory ASIC2-mediated calcineurin/NFAT against colorectal cancer by triterpenoids extracted from Rhus chinensis Mill. *J Ethnopharmacol* 235**,** 255-267.

Wang, G., Wang, Y.Z., Yu, Y., Wang, J.J., Yin, P.H., and Xu, K. (2020b). Triterpenoids Extracted from Rhus chinensis Mill Act Against Colorectal Cancer by Inhibiting Enzymes in Glycolysis and Glutaminolysis: Network Analysis and Experimental Validation. *Nutr Cancer* 72**,** 293-319.

Wang, G., Wang, Y.Z., Yu, Y., Yin, P.H., Xu, K., and Zhang, H. (2021). The Anti-Tumor Effect and Mechanism of Triterpenoids in Rhus chinensis Mill. on Reversing Effector CD8+ T-cells Dysfunction by Targeting Glycolysis Pathways in Colorectal Cancer. *Integr Cancer Ther* 20**,** 15347354211017219.

Wang, G., Yu, Y., Li, Z.M., Zhu, Z.M., Wang, Z.J., and Tao, M.F. (2022). Triterpenoids of Rhus chinensis Supressed Colorectal Cancer Progress by Enhancing Antitumor Immunity and CD8 + T Cells Tumor Infiltration. *Nutr Cancer* 74**,** 2550-2564.

Wang, L., Xu, J., Yan, Y., Liu, H., Karunakaran, T., and Li, F. (2019b). Green synthesis of gold nanoparticles from Scutellaria barbata and its anticancer activity in pancreatic cancer cell (PANC-1). *Artif Cells Nanomed Biotechnol* 47**,** 1617-1627.

Wang, M., Ma, C., Chen, Y., Li, X., and Chen, J. (2019c). Cytotoxic Neo-Clerodane Diterpenoids from *Scutellaria barbata* D.Don. *Chem Biodivers* 16**,** e1800499.

Wang, P., Li, Z., Fu, L., Zhu, J., Wu, X., Wang, Z., and Zhang, L. (2014). [Effects of extracts of Prunella Vulgaris L. on proteome of human lung adenocarcinoma cell line A549]. *Zhonghua Yi Xue Za Zhi* 94**,** 2216-2221.

Wang, Q., Acharya, N., Liu, Z., Zhou, X., Cromie, M., Zhu, J., and Gao, W. (2018). Enhanced anticancer effects of Scutellaria barbata D. Don in combination with traditional Chinese medicine components on non-small cell lung cancer cells. *J Ethnopharmacol* 217**,** 140-151.

Wei, L., Lin, J., Wu, G., Xu, W., Li, H., Hong, Z., and Peng, J. (2013). Scutellaria barbata D. Don induces G1/S arrest via modulation of p53 and Akt pathways in human colon carcinoma cells. *Oncol Rep* 29**,** 1623-1628.

Wei, L., Lin, J., Xu, W., Cai, Q., Shen, A., Hong, Z., and Peng, J. (2012). Scutellaria barbata D. Don inhibits tumor angiogenesis via suppression of Hedgehog pathway in a mouse model of colorectal cancer. *Int J Mol Sci* 13**,** 9419-9430.

Wei, L.H., Lin, J.M., Chu, J.F., Chen, H.W., Li, Q.Y., and Peng, J. (2017). Scutellaria barbata D. Don inhibits colorectal cancer growth via suppression of Wnt/β-catenin signaling pathway. *Chin J Integr Med* 23**,** 858-863.

Wei, P.Y., Pu, H.Q., Wei, X., Li, C.G., and Nong, S. (2007). Apoptosis-inducing effect of Scutellaria barbata extract on human lung cancer SPC-A-1 cells and the expression of apoptosis associated genes. *Zhong Yao Cai* 30**,** 1270-1273.

Widjaja, S.S., Rusdiana, and Ichwan, M. (2021). Enhanced cytotoxic effects of Clinacanthus nutans and doxorubicin in combination toward breast cancer cell lines. *J Adv Pharm Technol Res* 12**,** 152-156.

Wong, B.Y., Lau, B.H., Jia, T.Y., and Wan, C.P. (1996). Oldenlandia diffusa and Scutellaria barbata augment macrophage oxidative burst and inhibit tumor growth. *Cancer Biother Radiopharm* 11**,** 51-56.

Wong, B.Y., Nguyen, D.L., Lin, T., Wong, H.H., Cavalcante, A., Greenberg, N.M., Hausted, R.P., and Zheng, J. (2009). Chinese medicinal herb Scutellaria barbata modulates apoptosis and cell survival in murine and human prostate cancer cells and tumor development in TRAMP mice. *Eur J Cancer Prev* 18**,** 331-341.

Woo, H.J., Jun Do, Y., Lee, J.Y., Woo, M.H., Yang, C.H., and Kim, Y.H. (2011). Apoptogenic activity of 2α,3α-dihydroxyurs-12-ene-28-oic acid from Prunella vulgaris var. lilacina is mediated via mitochondria-dependent activation of caspase cascade regulated by Bcl-2 in human acute leukemia Jurkat T cells. *J Ethnopharmacol* 135**,** 626-635.

Wu, Z., Pan, X., Deng, C., Cai, M., Yuan, K., Huang, P., and Shi, G. (2022). Mechanism of Herb Pairs Astragalus mongholicus and Curcuma phaeocaulis Valeton in Treating Gastric Carcinoma: A Network Pharmacology Combines with Differential Analysis and Molecular Docking. *Evid Based Complement Alternat Med* 2022**,** 8361431.

Xu, C., Wang, Y., Feng, J., Qin, L., Xu, R., and Dou, Y. (2018). Effect of optimal combination of Huangqi (Radix Astragali Mongolici) and Ezhu (Rhizoma Curcumae Phaeocaulis) on proliferation and apoptosis of A549 lung cancer cells. *J Tradit Chin Med* 38**,** 351-358.

Xu, C., Wang, Y., Feng, J., Xu, R., and Dou, Y. (2019). Extracts from Huangqi (Radix Astragali Mongoliciplus) and Ezhu (Rhizoma Curcumae Phaeocaulis) inhibit Lewis lung carcinoma cell growth in a xenograft mouse model by impairing mitogen-activated protein kinase signaling, vascular endothelial growth factor production, and angiogenesis. *J Tradit Chin Med* 39**,** 559-565.

Xu, X., Chen, F., Zhang, L., Liu, L., Zhang, C., Zhang, Z., and Li, W. (2021). Exploring the mechanisms of anti-ovarian cancer of *Hedyotis diffusa* Willd and *Scutellaria barbata* D. Don through focal adhesion pathway. *J Ethnopharmacol* 279**,** 114343.

Xue, G.M., Xia, Y.Z., Wang, Z.M., Li, L.N., Luo, J.G., and Kong, L.Y. (2016). neo-Clerodane diterpenoids from Scutellaria barbata mediated inhibition of P-glycoprotein in MCF-7/ADR cells. *Eur J Med Chem* 121**,** 238-249.

Xue, S., Geng, A., Lian, T., and Liu, Y. (2022). *Scutellaria barbata* D. Don inhibits cervical cancer cell proliferation, migration, and invasion via miR-195-5p/LOXL2 axis. *Toxicol Res (Camb)* 11**,** 804-811.

Yakop, F., Abd Ghafar, S.A., Yong, Y.K., Saiful Yazan, L., Mohamad Hanafiah, R., Lim, V., and Eshak, Z. (2018). Silver nanoparticles Clinacanthus Nutans leaves extract induced apoptosis towards oral squamous cell carcinoma cell lines. *Artif Cells Nanomed Biotechnol* 46**,** 131-139.

Yang, J., Yang, G., Hou, G., Liu, Q., Hu, W., Zhao, P.U., and He, Y.I. (2015). Scutellaria barbata D. Don polysaccharides inhibit the growth of Calu-3 xenograft tumors via suppression of the HER2 pathway and angiogenesis. *Oncol Lett* 9**,** 2721-2725.

Yang, N., Zhao, Y., Wang, Z., Liu, Y., and Zhang, Y. (2017). Scutellarin suppresses growth and causes apoptosis of human colorectal cancer cells by regulating the p53 pathway. *Mol Med Rep* 15**,** 929-935.

Yang, P.W., Chen, T.T., Zhao, W.X., Liu, G.W., Feng, X.J., Wang, S.M., Pan, Y.C., Wang, Q., and Zhang, S.H. (2021). *Scutellaria barbata* D.Don and *Oldenlandia diffusa* (Willd.) Roxb crude extracts inhibit hepatitis-B-virus-associated hepatocellular carcinoma growth through regulating circRNA expression. *J Ethnopharmacol* 275**,** 114110.

Yang, X., Yang, Y., Tang, S., Tang, H., Yang, G., Xu, Q., and Wu, J. (2014a). Anti-tumor effect of polysaccharides from Scutellaria barbata D. Don on the 95-D xenograft model via inhibition of the C-met pathway. *J Pharmacol Sci* 125**,** 255-263.

Yang, X.K., Xu, M.Y., Xu, G.S., Zhang, Y.L., and Xu, Z.X. (2014b). In vitro and in vivo antitumor activity of scutebarbatine A on human lung carcinoma A549 cell lines. *Molecules* 19**,** 8740-8751.

Yang, Y., Fang, T., Cao, Y.L., Lv, Y.X., Chang, Q.Q., and Zhang, D.D. (2020). Ethyl Acetate Fraction from *Hedyotis diffusa* plus *Scutellaria barbata* Exerts Anti-Breast Cancer Effect via miR-200c-PDE7B/PD-L1-AKT/MAPK Axis. *Evid Based Complement Alternat Med* 2020**,** 3587095.

Yang, Z., Lu, S., Tang, H., Qu, J., Wang, B., Wang, Y., Pan, G., and Rao, B. (2022). Molecular Targets and Mechanisms of *Hedyotis diffusa*-*Scutellaria barbata* Herb Pair for the Treatment of Colorectal Cancer Based on Network Pharmacology and Molecular Docking. *Evid Based Complement Alternat Med* 2022**,** 6186662.

Ye, Z., Fang, Z., Li, D., Lin, X., and Huang, S. (2023). Exploring the material basis and mechanism of action of clinacanthus nutans in treating renal cell carcinoma based on metabolomics and network pharmacology. *Medicine (Baltimore)* 102**,** e35675.

Yeh, Y.C., Chen, H.Y., Yang, S.H., Lin, Y.H., Chiu, J.H., Lin, Y.H., and Chen, J.L. (2014). Hedyotis diffusa Combined with Scutellaria barbata Are the Core Treatment of Chinese Herbal Medicine Used for Breast Cancer Patients: A Population-Based Study. *Evid Based Complement Alternat Med* 2014**,** 202378.

Yin, D.T., Lei, M., Xu, J., Li, H., Wang, Y., Liu, Z., Ma, R., Yu, K., and Li, X. (2017). The Chinese herb Prunella vulgaris promotes apoptosis in human well-differentiated thyroid carcinoma cells via the B-cell lymphoma-2/Bcl-2-associated X protein/caspase-3 signaling pathway. *Oncol Lett* 14**,** 1309-1314.

Yin, X., Zhou, J., Jie, C., Xing, D., and Zhang, Y. (2004). Anticancer activity and mechanism of Scutellaria barbata extract on human lung cancer cell line A549. *Life Sci* 75**,** 2233-2244.

Yong, Y.K., Tan, J.J., Teh, S.S., Mah, S.H., Ee, G.C., Chiong, H.S., and Ahmad, Z. (2013). Clinacanthus nutans Extracts Are Antioxidant with Antiproliferative Effect on Cultured Human Cancer Cell Lines. *Evid Based Complement Alternat Med* 2013**,** 462751.

Yu, F., Zhang, L., Ma, R., Liu, C., Wang, Q., and Yin, D. (2021). The Antitumour Effect of Prunella vulgaris Extract on Thyroid Cancer Cells In Vitro and In Vivo. *Evid Based Complement Alternat Med* 2021**,** 8869323.

Yu, W.S., Jeong, S.J., Kim, J.H., Lee, H.J., Song, H.S., Kim, M.S., Ko, E., Lee, H.J., Khil, J.H., Jang, H.J., Kim, Y.C., Bae, H., Chen, C.Y., and Kim, S.H. (2011). The genome-wide expression profile of 1,2,3,4,6-penta-O-galloyl-β-D-glucose-treated MDA-MB-231 breast cancer cells: molecular target on cancer metabolism. *Mol Cells* 32**,** 123-132.

Zeng, S., Chen, L., Sun, Q., Zhao, H., Yang, H., Ren, S., Liu, M., Meng, X., and Xu, H. (2021). Scutellarin ameliorates colitis-associated colorectal cancer by suppressing Wnt/β-catenin signaling cascade. *Eur J Pharmacol* 906**,** 174253.

Zhang, J., Qi, C., Li, H., Ding, C., Wang, L., Wu, H., Dai, W., and Wang, C. (2023a). Exploration of the effect and mechanism of *Scutellaria barbata* D. Don in the treatment of ovarian cancer based on network pharmacology and in vitro experimental verification. *Medicine (Baltimore)* 102**,** e36656.

Zhang, K.J., Zhang, M.Z., Wang, Q.D., and Liu, W.L. (2006). [The experimental research about the effect of Prunella vulgaris L. on Raji cells growth and expression of apoptosis related protein]. *Zhong Yao Cai* 29**,** 1207-1210.

Zhang, L., Cai, Q., Lin, J., Fang, Y., Zhan, Y., Shen, A., Wei, L., Wang, L., and Peng, J. (2014). Chloroform fraction of Scutellaria barbata D. Don promotes apoptosis and suppresses proliferation in human colon cancer cells. *Mol Med Rep* 9**,** 701-706.

Zhang, L., Fang, Y., Feng, J.Y., Cai, Q.Y., Wei, L.H., Lin, S., and Peng, J. (2017a). Chloroform fraction of Scutellaria barbata D. Don inhibits the growth of colorectal cancer cells by activating miR‑34a. *Oncol Rep* 37**,** 3695-3701.

Zhang, L., Ren, B., Zhang, J., Liu, L., Liu, J., Jiang, G., Li, M., Ding, Y., and Li, W. (2017b). Anti-tumor effect of Scutellaria barbata D. Don extracts on ovarian cancer and its phytochemicals characterisation. *J Ethnopharmacol* 206**,** 184-192.

Zhang, M.Z., Sun, Z.C., Fu, X.R., Chen, C.Y., and Ding, M.J. (2009). [Study on proteomics of Jurkat cells treated with the extracts from Prunella vulgaris]. *Zhong Yao Cai* 32**,** 917-922.

Zhang, T., Gu, H.W., Gao, J.X., Li, Y.S., and Tang, H.B. (2022). Ethanol supernatant extracts of Gynura procumbens could treat nanodiethylnitrosamine-induced mouse liver cancer by interfering with inflammatory factors for the tumor microenvironment. *J Ethnopharmacol* 285**,** 114917.

Zhang, X., Shen, T., Zhou, X., Tang, X., Gao, R., Xu, L., Wang, L., Zhou, Z., Lin, J., and Hu, Y. (2020). Network pharmacology based virtual screening of active constituents of Prunella vulgaris L. and the molecular mechanism against breast cancer. *Sci Rep* 10**,** 15730.

Zhang, Z., Zhou, J., Guo, R., Zhou, Q., Wang, L., Xiang, X., Ge, S., and Cui, Z. (2023b). Network pharmacology to explore the molecular mechanisms of Prunella vulgaris for treating thyroid cancer. *Medicine (Baltimore)* 102**,** e34871.

Zheng, X., Kang, W., Liu, H., and Guo, S. (2018). Inhibition effects of total flavonoids from Sculellaria barbata D. Don on human breast carcinoma bone metastasis via downregulating PTHrP pathway. *Int J Mol Med* 41**,** 3137-3146.

Zheng, X.Q., Song, L.X., Han, Z.Z., Yang, Y.B., Zhang, Y., Gu, L.H., Yang, L., Chou, G.X., and Wang, Z.T. (2023). Pentacyclic triterpenoids from spikes of Prunella vulgaris L. with thyroid tumour cell cytostatic bioactivities. *Nat Prod Res* 37**,** 1518-1526.

Zhu, D., Yuan, S., and Chen, C. (2023a). Hedyotis diffusa-Sculellaria barbata (HD-SB) suppresses the progression of colorectal cancer cells via the hsa_circ_0039933/hsa-miR-204-5p/wnt11 axis. *Sci Rep* 13**,** 13331.

Zhu, J., Zhang, W., Zhang, Y., Wang, Y., Liu, M., and Liu, Y. (2018). Effects of Spica prunellae on caspase-3-associated proliferation and apoptosis in human lung cancer cells in vitro. *J Cancer Res Ther* 14**,** 760-763.

Zhu, X., Li, Y., Wang, X., Huang, Y., and Mao, J. (2023b). Investigation of the mechanism of Prunella vulgaris in treatment of papillary thyroid carcinoma based on network pharmacology integrated molecular docking and experimental verification. *Medicine (Baltimore)* 102**,** e33360.
